# Timing of an Adolescent Booster after Single Primary Meningococcal Serogroup C Conjugate Immunization at Young Age; An Intervention Study among Dutch Teenagers

**DOI:** 10.1371/journal.pone.0100651

**Published:** 2014-06-25

**Authors:** Susanne P. Stoof, Fiona R. M. van der Klis, Debbie M. van Rooijen, Mirjam J. Knol, Elisabeth A. M. Sanders, Guy A. M. Berbers

**Affiliations:** 1 Centre for Infectious Disease Control, National Institute of Public Health and the Environment (RIVM), Bilthoven, The Netherlands; 2 Department of Immunology and Infectious Diseases, Wilhelmina Children's Hospital, University Medical Center, Utrecht, The Netherlands; University of Missouri Kansas CIty School of Medicine, United States of America

## Abstract

**Background:**

Meningococcal serogroup C (MenC) specific antibody levels decline rapidly after a single primary MenC conjugate (MenCC) vaccination in preschool children. A second MenCC vaccination during (pre)adolescence might attain longer lasting individual and herd protection. We aimed to establish an appropriate age for a (pre)adolescent MenCC booster vaccination.

**Methods:**

A phase-IV trial with healthy 10-year-olds (n = 91), 12-year-olds (n = 91) and 15-year-olds (n = 86) who were primed with a MenCC vaccine nine years earlier. All participants received a booster vaccination with the same vaccine. Serum bactericidal antibody assay titers (SBA, using baby rabbit complement), MenC-polysaccharide (MenC-PS) specific IgG, IgG subclass and avidity and tetanus-specific IgG levels were measured prior to (T0) and 1 month (T1) and 1 year (T2) after the booster. An SBA titer ≥8 was the correlate of protection.

**Results:**

258 (96.3%) participants completed all three study visits. At T0, 19% of the 10-year-olds still had an SBA titer ≥8, compared to 34% of the 12-year-olds (P = 0.057) and 45% of the 15-year-olds (P<0.001). All participants developed high SBA titers (GMTs>30,000 in all age groups) and MenC-PS specific IgG levels at T1. IgG levels mainly consisted of IgG1, but the contribution of IgG2 increased with age. At T2, 100% of participants still had an SBA titer ≥8, but the 15-year-olds showed the highest protective antibody levels and the lowest decay.

**Conclusion:**

Nine years after primary MenCC vaccination adolescents develop high protective antibody levels in response to a booster and are still sufficiently protected one year later. Our results suggest that persistence of individual - and herd - protection increases with the age at which an adolescent booster is administered.

**Trial Registration:**

EU Clinical Trials Database 2011-000375-13 Dutch Trial Register NTR3521

## Introduction

Invasive meningococcal disease (IMD) is a severe illness, with a mortality rate of ∼10% and serious sequelae in a substantial number of survivors. In Europe serogroup B and C are responsible for the majority of disease [1]. A single Meningococcal Serogroup C conjugated (MenCC) vaccination was implemented into the Dutch National Immunization Programme (NIP) in 2002 for all children aged 14 months. In addition, a large catch-up campaign was conducted in 2002 during which all children between 1 and 19 years of age were invited to receive a MenCC vaccination. Total vaccine coverage of the catch-up campaign was 94% [2] whereas the current vaccine coverage for MenC in the NIP is >95% [3]. After 2002, MenC disease has almost completely disappeared. Thus far only three cases of vaccine failure have occurred, of which two had an underlying immune deficiency, and only a few cases of MenC disease occur yearly among unvaccinated individuals [4]. This successful elimination is likely due to the large-scale catch-up campaign that included adolescents [5], [6]. Teenagers and young adults have the highest nasopharyngeal meningococcal carriage levels and are considered the main source of spread [7]. As the MenCC vaccine not only protects against disease but also reduces nasopharyngeal carriage levels [8], large-scale vaccination of adolescents was very effective in preventing dissemination of MenC in the population [9].

In the past decade it became clear that MenC specific antibody levels wane rapidly in infants, even when primed with multiple doses [10], as well as in toddlers primed or boosted with a single dose [11]–[14]. The rapid decrease in quantitative antibody levels is associated with a fast reduction in the proportion of children with a serum bactericidal antibody (SBA) level – or protective antibody level - above the internationally accepted correlate of protection (≥8, when using baby rabbit complement). Different studies showed that the immunological memory response to MenC and the subsequent rise in protective antibody levels develop only after several days [15]–[18], whereas the meningococcus can invade the bloodstream and cause (fatal) disease within hours upon acquisition. Protective antibody levels above the correlate of protection are therefore crucial to maintain protection against MenC disease. Hence, priming young children with single or multiple MenCC vaccinations is not sufficient to maintain long term individual immunity. An additional MenCC vaccination at an older age may be required, especially since adolescents are also at particular risk for IMD. Primary vaccination in older children has led to higher and longer lasting protective antibody levels than in preschool children [12], [18]–[20]. Since adolescents are also considered as the main source of transmission, an additional vaccination prior to or during adolescence will prolong individual immunity but might also maintain the herd protection that has been achieved.

Studies investigating the response to a booster MenCC vaccination in adolescents showed promising results [19], [21] with good persistence of antibody levels in the following years [19], [20], [22]. However, these studies had relatively short intervals between the primary and booster vaccinations. To our knowledge, no studies exist that investigated the effect of an adolescent MenCC booster vaccination more than seven years after priming. To establish what would be an appropriate age for a (pre)adolescent booster, we recruited three age groups of 10-, 12- and 15-year-olds respectively who had received a primary MenCC vaccination nine years earlier. We investigated possible differences with age in MenC-specific SBA, IgG, IgG subclass and avidity levels at baseline and one month and one year after a booster MenCC vaccination.

## Methods

### Ethics Statement

This study was approved by the local ethics committee Verenigde Commissies Mensgebonden Onderzoek (VCMO, Nieuwegein, The Netherlands). The protocol for this trial and supporting TREND checklist are available as supporting information; see Protocol S1 and Checklist S1.

### Study design and participants

This study was a phase IV, single center, open-label study. In September 2011, an invitation letter was sent to parents of all children from Nieuwegein, Houten and Zeist (in the surrounding area of Utrecht, The Netherlands) within the targeted age range of 9.5–10.5 years, 11.5–12.5 years and 14.5–15.5 years (n = 4667). Inclusion criteria were good general health and a vaccination history according to the Dutch NIP, including one MenCC vaccination approximately nine years earlier (either during the catch-up campaign in 2002 or as part of the NIP) and the DT-IPV booster at 9 years [23]. Exclusion criteria were: severe acute illness/fever/use of antibiotics within 14 days prior to enrolment, disease/medical treatment that could interfere with results, allergy to vaccine component, serious adverse event after previous vaccination, previous meningococcal disease, multiple MenC vaccinations in immunization history, other vaccination within one month prior to enrolment, use of plasma products in the previous 6 months and pregnancy. Individual immunization history was verified by checking personal vaccination cards or from centralized immunization records. Written informed consent was obtained from both parents and participants aged ≥12 years prior to enrolment. The study was registered at the EU Clinical Trials database (EudraCT number: 2011-000375-13). Due to a communication error, this study was registered at the Dutch Trial register (www.trialregister.nl; NTR3521) after start of recruitment. The authors confirm that all ongoing and related trials for this intervention are registered.

### Clinical procedures

At the beginning of the study, participants received one vaccination with the MenC-polysaccharide conjugated to tetanus toxoid vaccine (MenC-TT, Baxter). Vaccinations were administered by intramuscular injection into the deltoid muscle, using a 23 gauge 0.6×25 mm needle. Blood samples (5 mL) were taken prior to the MenC-TT booster vaccination (T0) and 1 month (T1) and 1 year (T2) afterwards. Vaccinations and blood collections were performed by trained and authorized nurses at local study sites in Nieuwegein, Zeist and Houten. Blood samples were transported to the laboratory where sera were separated and stored at −20°C for serological analyses.

### Serological analyses

The level of MenC-specific protective antibodies was determined using the serum bactericidal antibody assay (SBA) with baby rabbit complement (Pelfreez, Rogers, AR) and target strain C11. SBA titers were expressed as the reciprocal of the final serum dilution yielding ≥50% killing at 60 minutes [24] with a titer of ≥8 as correlate of protection [25], [26].

MenC-polysaccharide (MenC-PS) specific IgG, IgG subclass, avidity and tetanus toxoid (TT) specific IgG levels were quantified using the fluorescent-bead-based multiplex immunoassay (MIA) as previously described [16], [27]–[29].

### Primary and secondary objectives

The primary objective was to determine potential differences between the age groups in SBA titers and proportion of participants with an SBA titer ≥8 and ≥128 prior to (T0) and 1 month (T1) and 1 year (T2) after the MenC-TT booster vaccination.

Secondary objectives included assessment of differences between groups in: 1) levels of serum MenC-PS specific IgG at T0; 2) persistence of vaccine induced MenC-PS specific IgG levels between T1 and T2; 3) MenC-PS specific IgG subclass and avidity levels at T0, T1 and T2; 4) serum IgG levels against tetanus toxoid (TT), the carrier protein for MenC polysaccharide in the conjugate vaccine.

### Statistical analysis

The study was powered to detect a twofold difference in SBA geometric mean titers (GMTs) between two of the age groups with and estimated SD of the log-transformed titer of 1.27 [19].

Proportion and 95% confidence intervals (95%CIs) of participants with an SBA titer ≥8 or ≥128 were calculated using the Agresti-Coull method [30]. Differences between groups in gender and proportion of participants with an SBA titer ≥8 and ≥128 were determined with a Chi-squared test.

GMTs of SBA, geometric mean concentrations (GMCs) of MenC-PS specific IgG, IgG subclass and TT-specific IgG, mean avidity and corresponding 95%CIs were calculated. Normal distribution of (log-transformed) values was checked prior to each analysis. Differences between groups in log-transformed titers and concentrations at the different time points were determined with linear regression analyses, adjusting for titers and concentrations at T0. Differences in mean avidity and GMC ratios were determined with paired and independent sample t-tests, respectively. Correlation between TT-specific IgG GMC at T0 and the difference between T0 and T2 was determined using Pearson correlation test. All p-values were adjusted for three comparisons with Bonferroni correction. A p-value ≤0.05 after correction was considered as statistically significant. Data were analyzed using Excel 2010 software (Microsoft Office) and SPSS statistics 19 (IBM).

## Results

### Study population

**Figure 1:**
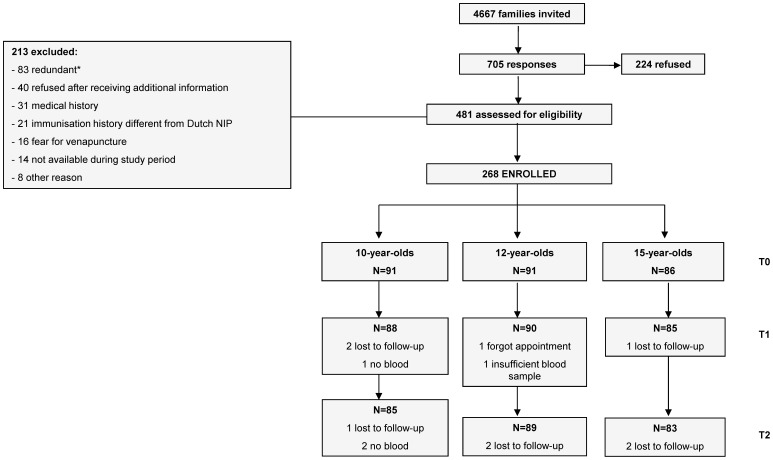
Flow-chart Recruitment. Flow chart for recruitment, enrolment and loss to follow-up. Participants were recruited in September 2011 from the surrounding area of Utrecht, The Netherlands. At the beginning of the study (T0) all participants received one booster vaccination with the Meningococcal serogroup C polysaccharide conjugated to tetanus toxoid vaccine (MenC-TT, Baxter); T1 and T2 indicate 1 month and 1 year after the MenC-TT booster respectively. *83 potential participants were excluded because the enrolment target of the study had been achieved.

In October 2011, 268 participants were enrolled and received the MenC-TT booster vaccination: a group with 10-year-olds (n = 91), a group with 12-year-olds (n = 91) and a group with 15-year-olds (n = 86). Blood samples of the 1 month (T1) and 1 year (T2) follow-up were collected in November 2011 and October 2012, respectively. 258 participants (96.3%) completed all three study visits (Figure 1). All participants were treated according to the protocol; analyses were performed on all available data.

Baseline characteristics are outlined in Table 1. There was no difference in gender between the groups. The 10-year-olds were primed at the age of 14 months according to the Dutch NIP whereas the 12- and 15-year-olds were primed during the catch-up campaign in 2002. There was a slight difference in interval since primary vaccination between the groups with the shortest interval for the 10-year-olds (8.7 years) and the longest for the 12-year-olds (9.3 years).

**Table 1:** Baseline Characteristics Study Population.

	10-year-olds	12-year-olds	15-year-olds
**No. of enrolled participants**	91	91	86
**Mean age at enrolment (T0)**; years (±SD)	9.9 (0.3)	12.0 (0.3)	15.0 (0.3)
**Female**; No. (%)	53 (58)	44 (48)	41 (48)
**Mean age at MenCC vaccine priming^a^**; years (±SD)	1.2 (0.1)	2.7 (0.3)	5.8 (0.4)
**Mean interval since primary MenCC vaccination and T0^b^**; years (±SD)	8.7 (0.3)	9.3 (0.1)	9.2 (0.2)

a. All participants were primed with one vaccination with the Meningococcal serogroup C polysaccharide conjugated to tetanus toxoid vaccine (MenC-TT, Baxter) and received a booster with the same vaccine at the beginning of the study (T0). Individual immunization histories were verified by checking personal vaccination cards or from centralized immunization records.

b. Intervals slightly differed between groups (separate t-tests; P<0.001). Further testing showed no relation between interval duration and antibody levels (data not shown). Analyses were therefore not adjusted for this interval.

### Primary objective

**Figure 2:**
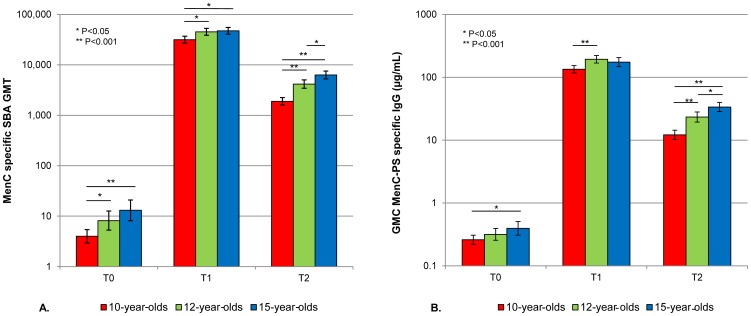
Meningococcal Serogroup C (MenC) Specific Geometric Mean Titers (GMTs) of Serum Bactericidal Antibody (SBA) and Geometric Mean Concentrations (GMCs) of MenC Polysaccharide (MenC-PS) specific Immunoglobulin G (IgG). MenC-specific GMTs of SBA (a) and GMCs of MenC-PS specific IgG (b) of different age groups prior to (T0) and 1 month (T1) and 1 year (T2) after the MenC conjugate booster.

At T0, nine years after the primary vaccination and prior to the MenC-TT booster vaccination, 17 (19%) of the 10-year-olds still had an SBA titer ≥8, compared to 31 (34%) of the 12-year-olds (P = 0.057) and 39 (45%) of the 15-year-olds (P<0.001; Table 2). In addition, 6 (7%) of the 10-year-olds had an SBA titer ≥128, compared to 16 (18%) of the 12-year olds (P = 0.069) and 23 (27%) of the 15-year olds (P<0.001; Table 2). Overall SBA GMTs were low, though values were slightly lower among the 10-year-olds compared to the 12-year-olds (P = 0.006) and the 15-year-olds (P<0.001; Figure 2a).

**Table 2:** Geometric Mean Titers (GMTs) of Meningococcal Serogroup C (MenC) Specific Serum Bactericidal Antibody (SBA) and Proportion of Participants with an SBA titer ≥8 and ≥128 prior to (T0) and 1 Month (T1) and 1 Year (T2) after the MenC Conjugate Booster.

			Age at T0		P-value difference between groups*		
		10 years	12 years	15 years	10 vs.12	10 vs. 15	12 vs. 15
**T0**	**GMT** (95%CI)	4.0 (2.9–5.4)	8.2 (5.3–12.6)	13.1 (8.1–21.0)	*0.006*	*<0.001*	0.459
	**SBA ≥8**: proportion	17/91	31/91	39/86			
	**SBA ≥8**: % (95%CI)	19 (12–28)	34 (25–45)	45 (35–56)	0.057	*<0.001*	0.375
	**SBA ≥128**: proportion	6/91	16/91	23/91			
	**SBA ≥128**: % (95%CI)	7 (3–14)	18 (11–27)	27 (18–37)	0.069	*<0.001*	0.426
**T1**	**GMT** (95%CI)	31,564 (26,899–37,038)	45,175 (38,608–52,859)	47,289 (40,422–55,322)	*0.021*	*0.003*	1.000
	**SBA ≥8**: proportion	88/88	89/89	85/85			
	**SBA ≥8**: % (95%CI)	100 (95–100)	100 (95–100)	100 (95–100)	1.000	1.000	1.000
	**SBA ≥128**: proportion	88/88	89/89	85/85			
	**SBA ≥128**: % (95%CI)	100 (95–100)	100 (95–100)	100 (95–100)	1.000	1.000	1.000
**T2**	**GMT** (95%CI)	1,987 (1,602–2,247)	4,165 (3,444–5,038)	6,292 (5,272–7,509)	*<0.001*	*<0.001*	*0.021*
	**SBA ≥8**: proportion	85/85	89/89	83/83			
	**SBA ≥8**: % (95%CI)	100 (95–100)	100 (95–100)	100 (95–100)	1.000	1.000	1.000
	**SBA ≥128**: proportion	85/85	89/89	83/83			
	**SBA ≥128**: % (95%CI)	100 (95–100)	100 (95–100)	100 (95–100)	1.000	1.000	1.000

**NOTE**: Differences between groups in SBA GMTs at T0 were determined using the Mann-Whitney U test. Differences between groups in SBA GMTs at T1 and T2 were determined with linear regression analyses, adjusting for titers at T0. An SBA titer ≥8 was considered as international correlate of protection. Differences between groups in proportion of participants with an SBA titer ≥8 and ≥128 was determined with χ^2^-tests.

* P-values were adjusted for three comparisons with Bonferroni correction. Extensive results of the crude and adjusted linear regression analyses are outlined in supplementary table S1.

At T1, one month after the MenC-TT booster vaccination, SBA GMTs had strongly increased to >30,000 in all age groups (Table 2, Figure 2a). SBA GMT in the 10-year-olds was lower compared to the 12-year-olds (P = 0.021) and the 15-year-olds (P = 0.003). All participants had an SBA titer ≥8 and ≥128 (Table 2).

At T2, one year after the MenC-TT booster vaccination, SBA GMTs had declined 16-fold in the 10-year-olds, 11-fold in the 12-year-olds and 8-fold in the 15-year-olds. SBA GMTs differed significantly between all groups (Figure 2a). Still, all participants had an SBA titer ≥8 and ≥128 (Table 2).

### Secondary objectives

#### MenC-PS specific IgG concentrations

Prior to the booster, the MenC-PS specific IgG GMCs were <0.5 µg/mL in all age groups, though slightly lower in the 10-year-olds compared to the 15-year-olds (P = 0.018; Table 3). In coherence with the SBA titers, GMCs of MenC-PS specific IgG had increased considerably at T1 in all age groups and decreased one year later. The level of decrease in IgG between T1 and T2 differed between all age groups and was the highest among the 10-year-olds and the lowest among the 15-year-olds (Table 3, Figure 2b).

**Table 3:** Geometric Mean Concentrations (GMCs) of Meningococcal Serogroup C Polysaccharide (MenC-PS) Specific Immunoglobulin G (IgG) prior to (T0) and 1 Month (T1) and 1 Year (T2) after the MenC Conjugate Booster.

			Age at T0		P-value difference between groups*		
		10 years	12 years	15 years	10 vs. 12	10 vs. 15	12 vs. 15
**T0**	**No. analyzed**	91	91	86			
	**GMC MenC-PS specific IgG** µg/mL (95%CI)	0.26 (0.22–0.31)	0.32 (0.26–0.39)	0.40 (0.31–0.51)	0.444	*0.018*	0.579
**T1**	**No. analyzed**	88	90	85			
	**GMC MenC-PS specific IgG** µg/mL (95%CI)	134 (117.0–153.4)	193.6 (168.2–222.3)	174.3 (147.5–206.0)	*<0.001*	0.063	1.000
**T2**	**No. analyzed**	85	89	83			
	**GMC MenC-PS specific IgG** µg/mL (95%CI)	12.2 (10.2–14.4)	23.3 (19.3–28.0)	33.7 (28.4–39.9)	*<0.001*	*<0.001*	*0.033*
**T1/T0**	**GMC ratio** (95%CI)	511 (417–627)	607 (469–787)	439 (322–598)	0.924	1.000	0.345
**T1/T2**	**GMC ratio** (95%CI)	11.0 (9.5–12.8)	8.3 (7.1–9.8)	5.2 (4.5–6.2)	*0.036*	*<0.001*	*<0.001*

**NOTE**: Differences between age groups in GMC of MenC-PS specific IgG were determined with linear regression analyses, adjusting for concentrations at T0. GMC ratios indicate the level of increase (T1/T0) or decrease (T1/T2) between time points. Differences in GMC ratios between groups were determined with independent sample t-tests.

* P-values were adjusted for three comparisons with Bonferroni correction. Extensive results of the crude and adjusted linear regression analyses are outlined in supplementary table S1.

#### MenC-PS specific IgG subclass concentrations

GMCs of MenC-PS specific IgG1 at T0 were equal in all age groups. The 10-year-olds showed a higher IgG1/IgG2 subclass ratio compared to the 12-year-olds (P = 0.027) and the 15-year-olds (P<0.001), which was due to a lower level of IgG2 compared to the other two groups. At T1 and T2, total MenC-PS specific IgG levels in all age groups mainly consisted of IgG1 subclass. The contribution of IgG2 remained the lowest in the 10-year-olds (Table 4).

**Table 4:** Geometric Mean Concentrations (GMCs) of Meningococcal Serogroup C Polysaccharide (MenC-PS) Specific Immunoglobulin G (IgG) Subclass and Subclass Ratio.

			Age at T0		P-value difference between groups*		
		10 years	12 years	15 years	10 vs. 12	10 vs. 15	12 vs. 15
**T0**	**No. analyzed**	91	91	86			
	**GMC IgG1** µg/mL (95%CI)	0.34 (0.29–0.41)	0.34 (0.27–0.44)	0.34 (0.27–0.43)	1.000	1.000	1.000
	**GMC IgG2** µg/mL (95%CI)	0.09 (0.08–0.11)^a^	0.13 (0.11–0.17)^b^	0.16 (0.12–0.21)	*0.024*	*0.003*	1.000
	**IgG1/IgG2**	3.98 (3.23–4.91)^a^	2.71 (2.23–3.30)^b^	2.17 (1.68–2.79)	*0.027*	*<0.001*	0.522
**T1**	**No. analyzed**	88	90	85			
	**GMC IgG1** µg/mL (95%CI)	158.6 (138.2–181.9)	213.4 (183.4–248.3)	168.7 (139.5–203.9)	*0.015*	1.000	0.174
	**GMC IgG2** µg/mL (95%CI)	14.0 (11.1–17.7)	28.0 (23.3–33.6)	30.5 (23.9–38.9)	*0.003*	*<0.001*	1.000
	**IgG1/IgG2**	11.30 (9.44–13.51)	7.59 (6.40–9.00)	5.54 (4.31–7.10)	0.255	0.054	0.276
**T2**	**No. analyzed**	85	89	83			
	**GMC IgG1** µg/mL (95%CI)	13.21 (11.32–15.42)	22.22 (18.51–26.68)	26.29 (22.39–30.88)	*<0.001*	*<0.001*	0.531
	**GMC IgG2** µg/mL (95%CI)	1.28 (1.02–1.59)	3.23 (2.54–4.12)	5.32 (3.99–7.10)	*<0.001*	*<0.001*	0.09
	**IgG1/IgG2**	10.36 (8.36–12.84)	6.87 (5.61–8.42)	4.94 (3.73–6.55)	0.969	0.156	0.297

**NOTE**: Differences between age groups in GMC of MenC-PS specific IgG subclass and subclass ratio were determined with linear regression analyses, adjusting for concentrations and ratios at T0.

* P-values were adjusted for three comparisons with Bonferroni correction. Extensive results of the crude and adjusted linear regression analyses are outlined in supplementary table S1.

a. Concentration IgG2 too low to measure in nine participants; GMC IgG2 and subclass ratio presented from 82 participants.

b. Concentration IgG2 too low to measure in five participants; GMC IgG2 and subclass ratio presented from 86 participants.

#### Avidity of MenC-PS specific IgG

**Figure 3:**
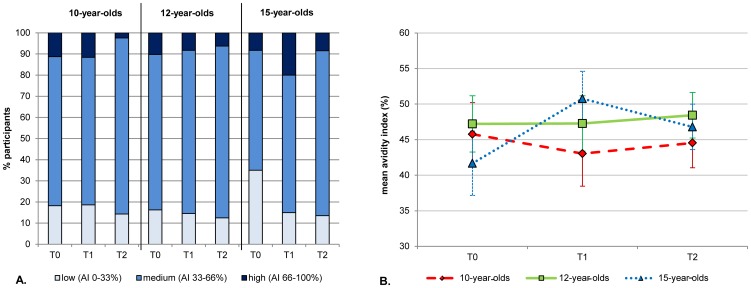
Meningococcal Serogroup C Polysaccharide Specific Immunoglobulin G (IgG) Avidity Indices (AI). Avidity indices divided in low, intermediate and high (a) and mean avidity indices (b) per age group prior to (T0) and 1 month (T1) and 1 year (T2) after the MenC conjugate booster. Only serum samples from participants with an IgG concentration of ≥0.25 µg/ml at T0 were included in the analyses (44 10-year-olds, 49 12-year-olds and 60 15-year-olds). Serum samples were diluted to a concentration of 25 ng/ml. Avidity index  =  (amount of IgG still bound after treatment with 0.5 M NH_4_SCN/amount of IgG with PBS) x 100.

Prior to the booster, mean avidity indices of MenC-PS specific IgG were similar between the three age groups (46%, 47% and 42% respectively). After the booster, avidity indices in the 10- and 12-year-olds remained at the same level. In contrast, the mean avidity index in the 15-year-olds increased to 51% at T1 and (P = 0.009) and subsequently decreased to 47% at T2 (P = 0.039; Figure 3).

#### Tetanus toxoid specific IgG concentrations

At T0, the GMC of TT-specific IgG was highest in the 10-year-olds and lowest in the 15-year-olds. One month after the MenC-TT booster, TT-specific IgG GMCs had increased in all age groups. The 10-year-olds showed the highest absolute concentration of antibody, but the level of increase was highest in the 15-year-olds. At T2, TT-specific IgG GMCs had decreased and remained highest in the 10-year-olds (Table 5). Overall, there was a negative correlation between the TT-specific IgG concentration at T0 and the difference in concentration between T0 and T2 (R = −0.694, P<0.001). A number of participants with a high concentration at T0, mostly 10-year-olds, showed a lower concentration at T2 and some even at T1.

**Table 5:** Geometric Mean Concentrations (GMCs) of Tetanus Toxoid (TT) Specific Immunoglobulin G (IgG) prior to (T0) and 1 Month (T1) and 1 Year (T2) after the Meningococcal Serogroup C Tetanus Toxoid Conjugate Booster.

			Age at T0		P-value difference between groups*		
		10 years	12 years	15 years	10 vs. 12	10 vs. 15	12 vs. 15
**T0**	**No. analyzed**	91	91	86			
	**GMC TT-specific IgG** µg/mL (95%CI)	5.1 (4.3–6.0)	1.6 (1.3–1.9)	0.7 (0.6–0.8)	*<0.001*	*<0.001*	*<0.001*
**T1**	**No. analyzed**	88	90	85			
	**GMC TT-specific IgG** µg/mL (95%CI)	7.4 (6.5–8.5)	4.4 (3.4–5.1)	2.9 (2.4–3.5)	0.069	0.213	1.000
**T2**	**No. analyzed**	85	89	83			
	**GMC TT-specific IgG** µg/mL (95%CI)	3.6 (3.1–4.2)	1.9 (1.6–2.2)	1.1 (1.0–1.4)	*0.006*	0.153	0.186
**T1/T0**	**GMC ratio** (95%CI)	1.49 (1.34–1.64)	2.75 (2.42–3.13)	4.26 (3.59–5.05)	*<0.001*	*<0.001*	*<0.001*
**T1/T2**	**GMC ratio** (95%CI)	2.03 (1.89–2.18)	2.34 (2.14–2.55)	2.50 (2.27–2.75)	*0.048*	*0.001*	0.921

**NOTE**: Differences between age groups in GMC of TT-specific IgG were determined with linear regression analyses, adjusting for concentrations at T0. GMC ratios indicate the level of increase (T1/T0) or decrease (T1/T2) between time points. Differences in GMC ratios between groups were determined with independent sample t-tests.

* P-values were adjusted for three comparisons with Bonferroni correction. Extensive results of the crude and adjusted linear regression analyses are outlined in supplementary table S1.

## Discussion

To our knowledge, this is the first study that investigated the immunological effect of an adolescent MenCC booster vaccination nine years after a single priming dose. Prior to the booster, the majority of participants had insufficient MenC-specific protective antibody levels. All participants developed high MenC-specific antibody levels one month after the booster. In addition, all participants were adequately protected one year later with 100% of the SBA titers ≥128. Of importance, the oldest age group (15-year-olds) maintained the highest antibody levels one year after the booster and showed the lowest level of antibody decrease.

The age of priming of the 10-, 12- and 15-year-olds in this study were 14 months, 2.8 years and 5.8 respectively. A Dutch serosurveillance study performed five years after the implementation of the MenCC vaccination in 2002, found that the percentage of children primed at these same ages with an SBA titer ≥8 were 47%, 39% and 50% respectively [12], [31]. At the beginning of our study and nine years after priming, the percentage of children with an SBA titer ≥8 had decreased to 19%, 34% and 45%, respectively. Clearly, the persistence of protective SBA titers after MenC primary vaccination is lower in children primed at 14 months compared to children primed at an older age. This corresponds with the established age-dependent increase in levels and persistence of MenC-specific antibody after primary vaccination [12], [20], [21]. A recent study from the UK reported protective SBA titers in only 15% of children primed ten years earlier with a single dose at age 1–4 years [32]. These findings clearly indicate that a single MenC conjugate vaccination at 14 months, as recommended in the Dutch NIP, is not sufficient to protect against MenC carriage and invasive disease during adolescence.

One month after the MenCC booster vaccination, all participants had developed very high antibody levels, despite differences in age at priming. Antibody levels in all age groups were higher than in comparable studies [18], [19]. An important difference with these studies is that all participants in our study were primed *and* boosted with a MenC-TT conjugate vaccine and not with the MenC-CRM197 conjugate vaccine. It has been shown that MenC-TT vaccines induce higher antibody responses after primary and booster vaccination and better persistence of antibody levels than MenC-CRM197 vaccines [11], [13], [33]–[35]. MenC-specific antibody levels at T1 were slightly lower in the 10-year-olds compared to the other two age groups. However, no difference between the age groups was found in the level of antibody increase one month following the booster (T1/T0 ratio). This suggests that the mechanism underlying the age-dependent differences in antibody levels after primary vaccination has limited influence on the antibody response to a booster when administered nine years later.

One year after the booster, the 15-year-olds showed the highest antibody levels and the lowest decay. Primary MenC conjugate vaccination during (pre)adolescence has been described to lead to long term persistence of protective SBA titers in >90% of individuals [12], [20]. A recent study from the UK reported even better persistence of SBA titers up to seven years after a booster dose given at the age of 13–15 years [20]. Of note, this booster was administered three years after the primary vaccination and the majority of participants still had protective SBA titers prior to the booster. Nevertheless, SBA-titers at one month and one year after the booster in this UK-study [22] were similar to our results. These findings suggest that a MenC booster administered at the age of 15 years could lead to persistence of protective antibody levels throughout young adulthood. It might even increase the transmission of antibodies from pregnant women to their babies, thereby prolonging protection of young infants and abrogating the need for infant priming. On the other hand, schoolchildren and younger adolescents might remain unprotected for a number of years if the booster is administered at an older age. However, since meningococcal carriage levels peak at approximately 19 years [7], a booster dose at 15 years is likely to prevent most transmission and thereby secure the herd protection. Interestingly, a recent model-based evaluation from Canada predicted that priming infants at 12 months and boosting adolescents at 15 years with a quadrivalent meningococcal conjugate vaccine against serogroups A, C, W and Y will be most effective at reducing incidence of meningococcal disease [36]. These results complement our findings and recommendation.

The high antibody levels after the booster were mostly caused by a rise in IgG1, indicating a T-cell dependent (TD) response. Another feature of TD responses is avidity maturation. Besides a small increase in avidity in the 15-year-olds one month after the booster, no clear increase in avidity was found in any of the groups one year after the booster. This suggests that maximal avidity has been achieved after primary vaccination. Data on avidity development after a (MenC) conjugate booster are scarce. Thus far, it has been shown that avidity increases with time following a primary MenC conjugate vaccination in infants and toddlers [35], [37], [38] and further increases one month after a polysaccharide or a conjugate booster have been described [35], [37], [39]. In contrast, a study in young adults showed high avidity one month after priming (regardless of the vaccine type) with no increase over time, nor - in the case of priming with polysaccharide vaccine - after a booster dose of conjugate vaccine. [40]. Hence, the absence of avidity increase in our study might be an age-related rather than a booster-related phenomenon. Interestingly, we recently reported decreasing avidity indices with increasing age in the cohort primed five years earlier during the catch-up campaign [38]. This reduction in avidity was related to increasing levels of MenC-specific IgG2 with age. In the current study, we also found increasing levels of IgG2 with age, moderate levels of avidity of MenC-specific antibodies overall and a higher proportion of 15-year-olds with low avidity prior to the booster. Our current results therefore underline the suggestion that the immune response to a conjugate vaccine at ages above infancy is not entirely T-cell dependent but also shows increasing features of a T-cell independent response with age [38].

The Dutch NIP offers children a final DT-IPV booster at the age of 9 years. Consequently, TT-specific IgG levels at T0 were highest in the 10-year-olds. After the MenC-TT booster, TT-specific GMCs increased in all age groups. However, a number of participants with a high concentration at T0 – mostly 10-year-olds - showed only a small increase in concentration at T1 and T2. Some 10-year-olds even showed a decrease. It is possible that the newly administered tetanus toxoid was cleared by the high levels of pre-existing antibodies in these individuals. Although the TT-specific GMCs remained far above the international protective threshold of 0.1 IU/mL, this extra MenC-TT booster was not very beneficial for these individuals in terms of tetanus immunity. Therefore, postponing an adolescent MenC-TT booster up to a few years after the last routine tetanus booster seems more beneficial in the long term with regards to protection against tetanus.

This study has important strengths, such as the longitudinal study design, adequate sample size and small loss to follow-up. Furthermore, all participants were primed and boosted with a single dose of the same vaccine. Finally, the 10-year-olds represented children primed according to the current NIP. Limitations of this study were the short follow-up period and that additional demographic information other than age and sex was not collected. Furthermore, participants were primed at different ages. To what extent the latter has influenced the antibody response to the booster is unclear. High antibody levels in all participants at T1 and the absence of significant differences between the age groups in the level of antibody increase in the first month after the booster, suggests that the influence of the age at priming on the memory response was minor. However, we cannot be certain that we would observe similar antibody responses in 15-year-olds primed at 14 months. The proportion of 10-year-olds in this study, all primed at 14 months, with an SBA titer <8 at T0 was >80%. Therefore, it is to be expected that virtually all individuals primed at 14 months will have an SBA titer<8 by the time they are 15. To anticipate for this, we performed a sub analysis including only participants with an SBA titer <8 (i.e. not protected) at T0. We found similar antibody levels following the booster and similar differences between the age groups in antibody decay as when total groups were analyzed (data not shown).

To conclude, nine years after their primary MenCC vaccination, adolescents develop considerably high antibody levels in response to a MenCC booster vaccination and are still sufficiently protected one year later. The oldest age group in this study maintained the highest (protective) antibody levels one year after the booster and showed the lowest level of antibody decrease. This suggests that persistence of individual - and indirect herd - protection increases with the age at which an adolescent MenCC booster is administered.

## Supporting Information

Table S1 **Results of crude and adjusted linear regression analyses for differences between age groups in Meningococcal serogroup C specific SBA geometric mean titers (GMTs) and geometric mean concentrations (GMCs) of IgG, IgG1, IgG2, IgG1/IgG2 ratio and tetanus toxoid (TT)-specific IgG.**
(DOCX)

Protocol S1 **Study protocol as approved by the local ethics committee Verenigde Commissies Mensgebonden Onderzoek (VCMO, Nieuwegein, The Netherlands).**
(PDF)

Checklist S1 **TREND checklist for standardized reporting of this non-randomized controlled trial.**
(XLSX)
