# Supplementary material for: Timing of an Adolescent Booster after Single Primary Meningococcal Serogroup C Conjugate Immunization at Young Age; An Intervention Study among Dutch Teenagers
Source: PLoS One. 2014 Jun 25;9(6):e100651. doi: 10.1371/journal.pone.0100651 (PMC4070982; doi:10.1371/journal.pone.0100651)
Supplement: Table S1 — Results of crude and adjusted linear regression analyses for differences between age groups in Meningococcal serogroup C specific SBA geometric mean titers (GMTs) and geometric mean concentrations (GMCs) of IgG, IgG1, IgG2, IgG1/IgG2 ratio and tetanus toxoid (TT)-specific IgG. (DOCX) [file pone.0100651.s001.docx]

**Table S1. Results of crude and adjusted linear regression analyses for differences between age groups in Meningococcal serogroup C specific SBA geometric mean titers (GMTs) and geometric mean concentrations (GMCs) of IgG, IgG1, IgG2, IgG1/IgG2 ratio and tetanus toxoid (TT)-specific IgG.**

|  |  | **10 vs. 12** |  |  | **10 vs. 15** |  |  | **12 vs. 15** |  |
| --- | --- | --- | --- | --- | --- | --- | --- | --- | --- |
| **T0 (prior to MenC-TT booster)** | **GMC ratio*** | **95% CI** | **P-value**** | **GMC ratio*** | **95% CI** | **P-value**** | **GMC ratio*** | **95% CI** | **P-value**** |
| **SBA** | n.a. | n.a. | n.a. | n.a. | n.a. | n.a. | n.a. | n.a. | n.a. |
| **MenC-PS specific IgG** | 1.23 | 0.93-1.61 | 0.444 | 1.53 | 1.13-2.06 | *0.018* | 1.24 | 0.89-1.73 | 0.579 |
| **MenC-PS specific IgG1** | 1 | 0.74-1.36 | 1 | 1 | 0.75-1.33 | 1 | 1 | 0.71-1.40 | 1 |
| **MenC-PS specific IgG2** | 1.46 | 1.11-1.92 | *0.024* | 1.72 | 1.23-2.39 | *0.003* | 1.18 | 0.82-1.70 | 1 |
| **MenC-PS specific IgG1/IgG2** | 0.68 | 0.51-0.91 | *0.027* | 0.54 | 0.39-0.76 | *<0.001* | 0.8 | 0.58-1.11 | 0.522 |
| **TT-specific IgG** | 0.32 | 0.25-0.41 | *<0.001* | 0.13 | 0.10-0.17 | *<0.001* | 0.42 | 0.32-0.54 | *<0.001* |
| **T1 (one month after MenC-TT booster)** | **GMC ratio*** | **95% CI** | **P-value**** | **GMC ratio*** | **95% CI** | **P-value**** | **GMC ratio*** | **95% CI** | **P-value**** |
| **SBA^a^** | 1.43 | 1.14-1.79 | *0.006* | 1.5 | 1.19-1.88 | *0.003* | 1.05 | 0.84-1.31 | 1 |
| **SBA ^b^** | 1.37 | 1.09-1.72 | *0.021* | 1.52 | 1.20-1.93 | *0.003* | 1.04 | 0.83-1.30 | 1 |
| **MenC-PS specific IgG^a^** | 1.44 | 1.19-1.76 | *<0.001* | 1.3 | 1.05-1.61 | 0.051 | 0.9 | 0.72-1.12 | 1 |
| **MenC-PS specific IgG^b^** | 1.44 | 1.18-1.75 | *<0.001* | 1.3 | 1.04-1.62 | 0.063 | 0.9 | 0.73-1.13 | 1 |
| **MenC-PS specific IgG1^a^** | 1.35 | 1.10-1.65 | *0.015* | 1.06 | 0.84-1.34 | 1 | 0.79 | 0.62-1.01 | 0.174 |
| **MenC-PS specific IgG1^b^** | 1.35 | 1.09-1.66 | *0.015* | 1.06 | 0.84-1.34 | 1 | 0.79 | 0.62-1.01 | 0.174 |
| **MenC-PS specific IgG2^a^** | 1.99 | 1.48-2.69 | *<0.001* | 2.17 | 1.54-3.05 | *<0.001* | 1.09 | 0.80-1.48 | 1 |
| **MenC-PS specific IgG2^b^** | 1.72 | 1.26-2.35 | *0.003* | 1.68 | 1.20-2.36 | *<0.001* | 1.02 | 0.76-1.37 | 1 |
| **MenC-PS specific IgG1/IgG2^a^** | 0.67 | 0.52-0.86 | *0.006* | 0.49 | 0.36-0.67 | *<0.001* | 0.73 | 0.54-0.99 | 0.12 |
| **MenC-PS specific IgG1/IgG2^b^** | 0.81 | 0.63-1.03 | 0.255 | 0.74 | 0.58-0.95 | 0.054 | 0.81 | 0.64-1.03 | 0.276 |
| **TT-specific IgG^a^** | 0.59 | 0.48-0.73 | *<0.001* | 0.39 | 0.31-0.49 | *<0.001* | 0.65 | 0.51-0.83 | *0.003* |
| **TT-specific IgG^b^** | 1.21 | 1.03-1.42 | 0.069 | 1.26 | 0.98-1.63 | 0.213 | 1.07 | 0.88-1.31 | 1 |
| **T2 (one year after MenC-TT booster)** | **GMC ratio*** | **95% CI** | **P-value**** | **GMC ratio*** | **95% CI** | **P-value**** | **GMC ratio*** | **95% CI** | **P-value**** |
| **SBA^a^** | 2.19 | 1.70-2.84 | *<0.001* | 3.32 | 2.59-4.24 | *<0.001* | 1.51 | 1.16-1.96 | *0.006* |
| **SBA^b^** | 1.98 | 1.55-2.54 | *<0.001* | 3.06 | 2.36-3.95 | *<0.001* | 1.43 | 1.11-1.84 | *0.021* |
| **MenC-PS specific IgG^a^** | 1.92 | 1.48-2.47 | *<0.001* | 2.77 | 2.17-3.53 | *<0.001* | 1.45 | 1.12-1.86 | *0.015* |
| **MenC-PS specific IgG^b^** | 1.82 | 1.43-2.33 | *<0.001* | 2.55 | 2.01-3.24 | *<0.001* | 1.37 | 1.07-1.75 | *0.033* |
| **MenC-PS specific IgG1^a^** | 1.68 | 1.32-2.14 | *<0.001* | 1.99 | 1.59-2.49 | *<0.001* | 1.18 | 0.92-1.51 | 0.54 |
| **MenC-PS specific IgG1^b^** | 1.69 | 1.35-2.13 | *<0.001* | 1.99 | 1.59-2.48 | *<0.001* | 1.17 | 0.93-1.49 | 0.531 |
| **MenC-PS specific IgG2^a^** | 2.54 | 1.82-3.54 | *<0.001* | 4.17 | 2.89-6.01 | *<0.001* | 1.64 | 1.13-2.40 | *0.03* |
| **MenC-PS specific IgG2^b^** | 2.16 | 1.55-3.01 | *<0.001* | 3.06 | 2.16-4.33 | *<0.001* | 1.45 | 1.04-2.03 | 0.09 |
| **MenC-PS specific IgG1/IgG2^a^** | 0.66 | 0.49-0.89 | *0.021* | 0.48 | 0.33-0.68 | *<0.001* | 0.72 | 0.51-1.17 | 0.186 |
| **MenC-PS specific IgG1/IgG2^b^** | 0.87 | 0.67-1.14 | 0.969 | 0.76 | 0.58-1.00 | 0.156 | 0.81 | 0.63-1.04 | 0.297 |
| **TT-specific IgG^a^** | 0.52 | 0.42-0.65 | *<0.001* | 0.32 | 0.25-0.40 | *<0.001* | 0.6 | 0.48-0.77 | *<0.001* |
| **TT-specific IgG^b^** | 1.22 | 1.08-1.39 | *0.006* | 1.24 | 1.00-1.54 | 0.153 | 1.15 | 0.99-1.33 | 0.186 |

**NOTE:** *GMC ratio is equal to exponent of regression coefficient (β) from linear regression analysis on log-transformed values; for the serum bactericidal antibody assay (SBA) results, the GMT ratio is presented. ** P-value adjusted for three comparisons with Bonferroni correction. n.a.= not applicable, log-transformed SBA titers at T0 were not normally distributed; differences in SBA titers at T0 between groups were calculated using the Mann-Whitney U test.

a. model 1: crude analysis; b. model 2: analysis adjusted for values at T0.
