# Supplementary material for: Timing of an Adolescent Booster after Single Primary Meningococcal Serogroup C Conjugate Immunization at Young Age; An Intervention Study among Dutch Teenagers
Source: PLoS One. 2014 Jun 25;9(6):e100651. doi: 10.1371/journal.pone.0100651 (PMC4070982; doi:10.1371/journal.pone.0100651)
Supplement: Protocol S1 — Study protocol as approved by the local ethics committee Verenigde Commissies Mensgebonden Onderzoek (VCMO, Nieuwegein, The Netherlands). (PDF) [file pone.0100651.s002.pdf]

APPROPRIATE AGE FOR SECOND IMMUNIZATION MEN C STUDY

(In Dutch: ‘**T**weede **I**mmunisatie **M**en C (**TIM)**-studie’**)**

Study to determine the appropriate age for a second Meningococcal serogroup C conjugate vaccination

Research protocol Version 8 September 9, 2011

PROTOCOL TITLE

Second Immunization MenC study (Dutch acronym: TIM-studie);

Study to determine the appropriate age for a second Meningococcal serogroup C conjugate vaccination

**Protocol ID**	**Protocol LIS-144; TIM-study**
**Short title**	**Appropriate age for second immunization MenC study**
**Version**	**08**
**Date**	**September, 2011**
**Coordinating investigator/project leader**	**Guy Berbers, PhD (Clb, RIVM)**<br>**RIVM**<br>**PO Box 1**<br>**3720 BA Bilthoven**<br>**Tel: +31 (0)30-274 2496**<br>**E-mail: Guy.Berbers@RIVM.nl**
**Principal investigator(s) (in Dutch: hoofdonderzoeker/uitvoerder)**	**1. Susanne Stoof, PhD-student**<br>**Tel: +31 (0)30-274 3317**<br>**E-mail: Susanne. Stoof@RIVM.nl**<br>**2. Fiona van der Klis, PhD**<br>**Tel: +31 (0)30-274 2272**<br>**E-mail: Fiona.van.der.Klis@RIVM.nl**<br>**3. Lieke Sanders, MD, PhD**<br>**Tel: +31 (0)30-274 2496**<br>**E-mail: L.Sanders@umcutrecht.nl**
**Sponsor (in Dutch: verrichter/opdrachtgever)**	**National Institute for Public Health and Environment (RIVM)**<br>**Antonie van Leeuwenhoeklaan 9**<br>**3721 MA Bilthoven**<br>**Postbus 1**<br>**3720 BA Bilthoven**
**Independent physician(s)**	**Tom Wolfs, MD, PhD**<br>**Tel: +31 (0)30-274 4980**<br>**E-mail: T.Wolfs@umcutrecht.nl**

**Laboratory sites**	**National Institute for Public Health and the Environment (RIVM)**<br>**Centre for Infectious Disease Control (CIb)**<br>**Laboratory for Infectious Diseases and Screening (LIS)**<br>**Antonie van Leeuwenhoeklaan 9**<br>**3721 MA Bilthoven**

PROTOCOL SIGNATURE SHEET

Name	Signature	Date
**Sponsor or legal representative:**<br>**National Institute for Public Health and Environment *(RIVM),***<br>**Centre for Infectious Disease Control, *(CIb).***<br><br>**For non-commercial research,**<br>**Head of Department:**<br>**Prof. dr. R.A. Coutinho**		
**Coordinating Investigator/Project leader/**<br>**Dr. W.A.M. Berbers (CIb, RIVM)**		
**Principal Investigator 1:**<br>**Drs. S.P.Stoof (CIb, RIVM)**		
**Principal Investigator 2:**<br>**Dr. F.R. van der Klis (CIb, RIVM)**		
**Principal Investigator 3:**<br>**Prof. Dr. E.A.M. Sanders (WKZ, UMCU)**		

TABLE OF CONTENTS

1. INTRODUCTION AND RATIONALE 10
2. OBJECTIVES 14
3. STUDY DESIGN 15
4. STUDY POPULATION 17
4.1 Population (base) 17
4.2 Inclusion criteria 17
4.3 Exclusion criteria 17
4.4 Sample size calculation 18
5. TREATMENT OF SUBJECTS 20
5.1 Investigational product/treatment 20
5.2 Use of co-intervention 20
5.3 Escape medication 20
6. INVESTIGATIONAL MEDICINAL PRODUCT 21
6.1 Name and description of investigational medicinal product 21
6.2 Summary of findings from non-clinical studies 21
6.3 Summary of findings from clinical studies 21
6.4 Summary of known and potential risks and benefits 21
6.5 Description and justification of route of administration and dosage 21
6.6 Dosages, dosage modifications and method of administration 21
6.7 Preparation and labelling of Investigational Medicinal Product 21
6.8 Drug accountability 21
7. METHODS 22
7.1 Study parameters/endpoints 22
7.1.1 Main study parameters 22
7.1.2 Secondary study parameters 22
7.1.3 Other study parameters 23
7.2 Randomisation, blinding and treatment allocation 23
7.3 Study procedures 23
7.4 Withdrawal of individual subjects 24
7.5 Replacement of individual subjects after withdrawal 24
7.6 Follow-up of subjects withdrawn from treatment 24
7.7 Premature termination of the study 24
8. SAFETY REPORTING 26
8.1 Section 10 WMO event 26
8.2 Adverse and serious adverse events 26
8.2.1 Suspected unexpected serious adverse reactions (SUSAR) 27
8.2.2 Annual safety report 27
8.3 Follow-up of adverse events 28
8.4 Data Safety Monitoring Board (DSMB) 28
9. STATISTICAL ANALYSIS 29
9.1 Descriptive statistics 29

9.2 Univariate analysis 29
9.3 Multivariate analysis 30
9.4 Interim analysis 30
10. ETHICAL CONSIDERATIONS 31
10.1 Regulation statement 31
10.2 Recruitment and informed consent 31
10.3 Objection by minors 32
10.4 Benefits and risks assessment 32
10.5 Compensation for injury 32
10.6 Incentives 32
11. ADMINISTRATIVE ASPECTS AND PUBLICATION 33
11.1 Handling and storage of data and documents 33
11.2 Amendments 33
11.3 Annual progress report 34
11.4 End of study report 34
11.5 Public disclosure and publication policy 34
12. REFERENCES 35

LIST OF ABBREVIATIONS AND RELEVANT DEFINITIONS

ABR	ABR form, General Assessment and Registration form, is the application form that is required for submission to the accredited Ethics Committee (In Dutch, ABR = Algemene Beoordeling en Registratie)
AE	Adverse Event
AR	Adverse Reaction
CA	Competent Authority
CCMO	Central Committee on Research Involving Human Subjects; in Dutch: Centrale Commissie Mensgebonden Onderzoek
CV	Curriculum Vitae
DSMB	Data Safety Monitoring Board
EU	European Union
EudraCT	European drug regulatory affairs Clinical Trials
GCP	Good Clinical Practice
IB	Investigator's Brochure
IC	Informed Consent
IMP	Investigational Medicinal Product
IMPD	Investigational Medicinal Product Dossier
MenC-PS	Meningococcal serogroup C polysaccharide
MenCC vaccine	Meningococcal serogroup C conjugated vaccine
METC	Medical research ethics committee (MREC); in Dutch: medisch ethische toetsing commissie (METC)
NIP	National Immunization Programme (in Dutch: Rijks Vaccinatie Programma, RVP)
(S)AE	(Serious) Adverse Event
SBA	Serum Bactericidal Antibody Assay
SPC	Summary of Product Characteristics (in Dutch: officiële productinfomatie IB1-tekst)
Sponsor	The sponsor is the party that commissions the organisation or performance of the research, for example a pharmaceutical company, academic hospital, scientific organisation or investigator. A party that provides funding for a study but does not commission it is not regarded as the sponsor, but referred to as a subsidising party.
SUSAR	Suspected Unexpected Serious Adverse Reaction
Wbp	Personal Data Protection Act (in Dutch: Wet Bescherming Persoonsgevens)
WMO	Medical Research Involving Human Subjects Act (in Dutch: Wet Medisch-wetenschappelijk Onderzoek met Mensen

SUMMARY

Rationale: In 2002 a Meningococcal serogroup C conjugated (MenCC) vaccination was implemented into the Dutch National Immunization Programme (NIP) for all children aged 14 months. In addition, a catch-up campaign was conducted between June and November 2002 during which all children between 1 and 18 years were invited to receive a single MenCC vaccination. Overall vaccine coverage was 94% and afterwards MenC disease disappeared in the vaccinated cohorts and even decreased dramatically in the non-immunized cohorts. It is suggested that the great success of the MenCC vaccination is primarily based on the catch-up campaign inducing large scale herd immunity by reducing the nasopharyngeal carriage of MenC bacteria in the population.

Available data derived from studies in the Netherlands and the UK now show that it might be necessary to introduce a second MenCC vaccine immunization in the NIP in order to maintain long-term individual and herd immunity against MenC. MenC-polysaccharide (MenC-PS) specific antibody levels decline rapidly after primary vaccination in young children. Protection induced by a primary MenCC vaccination appears to be age-dependant: cohorts vaccinated at older ages (up to adolescence/early adult) reveal greater and longer lasting protection than those routinely vaccinated in infancy. Next to an increased risk of invasive MenC disease in young children, there is an increased risk of invasive MenC disease during the teenage years. This suggests that a second MenCC vaccination may be needed to maintain the successful contribution this vaccine has made to public (child) health in the Netherlands. Without a second dose of MenCC vaccine at an older age, children vaccinated at 14 months will reach the second period of increased risk for invasive MenC disease with low serologic markers of protective immunity.

Objective: To determine the appropriate age for a second MenCC vaccination.

Study design: Intervention study.

Study population: Participants eligible to this study are healthy Dutch children that received all regular vaccinations according to the NIP. Three age-groups will be created: 10-, 12- and 15 year olds; n=82 per group. All children must have received a primary MenCC vaccination at an earlier age, either during the mass catch-up campaign in 2002 (12- and 15 years olds) or at the age of 14 months (regular vaccination time point as part of the NIP; 10-year olds).

Intervention: Participants will receive one injection with the MenC conjugated vaccine that is registered and used in the Dutch NIP (NeisVac-C™; 0,5 mL) intramuscularly in the upper arm. Blood and saliva samples will be taken prior to and 1 month and 1 year after the vaccination.

Main study parameters: Serum MenC-PS specific IgG antibodies and serum bactericidal antibody assay (SBA) levels.

Nature and extent of the burden and risks associated with participation, benefit and group relatedness: Participants benefit from participating in the study by receiving an additional MenCC vaccination. From the public health perspective, participation in this study will contribute to the improvement of the National Immunisation Programme (NIP). Vaccination and venapunctures might be painful and unpleasant. On request of the participant, Xylocainespray can be used to reduce possible local pain during the venapunction. NeisVac-C™ is a registered vaccine in the Netherlands. Mild adverse reactions to the vaccine may occur but they are expected to be mainly local and

transient. Severe allergic reactions to one of the vaccine components are unlikely to occur. As a compensation for the vaccination and the venapunctures, all participants will receive a total of €25,- in vouchers.

1. INTRODUCTION AND RATIONALE

Neisseria meningitides is a gram-negative diplococcal bacterium that is carried in the nasopharynx by 8-25% of the human population (1). Assessment of tonsillar tissue after tonsillectomy reveals even higher carriage rates up to 45%(2). In Europe and North America, carriage rates are low in the first years of life, and then sharply increase in teenagers, reaching a peak in those aged between 20 and 24 years (3). Transmission mainly occurs via respiratory droplets and saliva. As the bacterium is a commensal, it mostly does not cause symptoms and disappears after several days to months. However, acquisition of N. meningitides can also lead to local inflammation, invasion of mucosal surfaces, access to the bloodstream and development of rapidly progressive meningitis and/or sepsis (1). Although N. meningitides is susceptible for antibiotics, morbidity (hearing loss, scarring and amputation of limbs) and mortality rates of invasive disease remain high. The mortality rate for invasive meningococcal disease is approximately 10% and in case of severe sepsis even higher (4).

N. meningitides is a strictly human pathogen. Based on the capsular groups, 13 different serotypes of N. meningitides have been identified, but only six (A,B,C,W-135, X and Y) are associated with invasive disease (1). Serogroup B and C are responsible for most cases of invasive disease in industrialized European countries including the Netherlands (5).

Despite high carriage levels the incidence of disease caused by Meningococcal serogroup C (MenC) in the Netherlands was generally low in the past, with children in the age-groups 1-5 and 12-18 years usually showing the highest disease incidence. In 1995/1996 the incidence of MenC disease in the Netherlands was approximately 0.35 per 100.000 inhabitants. However, by the end of the 90's the incidence of MenC disease suddenly increased throughout Europe. In 2000/2001 the incidence of MenC disease in the Netherlands had increased to 1.17 per 100.000 inhabitants. This led to vast media attention, increasing public anxiety and the governmental decision to include a MenC vaccination into the Dutch National Immunization Programme (NIP). In September 2002, a single Men C conjugate protein-capsular polysaccharide vaccination (MenCC, Neisvac-C, Baxter, IL, USA) at the age of 14 months was introduced into the Dutch NIP. In addition, a catch-up campaign was conducted between June and November 2002 during which all children between 1 and 18 years were invited to receive a single MenCC vaccination. Overall vaccine coverage was 94% and afterwards MenC disease disappeared in the vaccinated cohorts and even decreased dramatically in the non-immunized cohorts. Interestingly, the decrease in MenC disease was also paralleled by a decrease in Meningococcal serogroup B disease for which no vaccine is currently available yet (figure 1) (6).

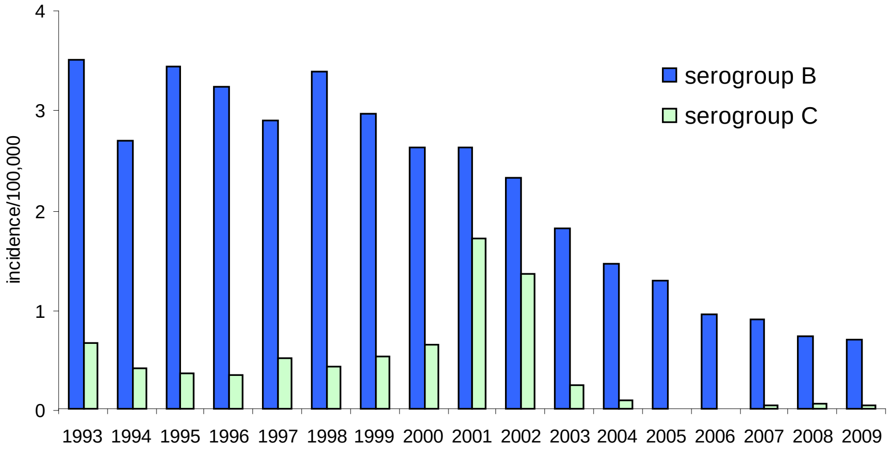

Fig. 1: Incidence of Meningococcal B and C disease in the Netherlands.

Similar results were obtained in the UK which was the first country to introduce MenCC vaccination. The MenCC vaccination was implemented into the UK NIP in 1999 as a 3-doses vaccination-schedule at the age of 2, 3 and 4 months (this schedule has been changed in 2006 to 2+1 schedule at 3, 4 and 12 months). In addition, a catch-up campaign was also conducted in 1999 in children aged between 1-18 years. Vaccine coverage was >90% in routine infant immunization and 85% in the catch-up campaign (7). After introduction, the incidence of MenC disease decreased substantially in both immunised *and* unimmunised individuals (8). It is suggested that this great success of the MenCC vaccination is primarily based on the catch-up campaign inducing large scale herd immunity by reducing the nasopharyngeal carriage of MenC bacteria in the population. The present low incidence of MenC disease is considered to be a consequence of herd-immunity and to a much lesser degree to individual immunity (9). Sustained immunization of a large part of the population should therefore be pursued.

Unfortunately, it became clear recently that vaccine-induced immunity after primary immunization is not sustained in young children (10-12). MenC-PS specific IgG antibody concentrations decline rapidly after vaccination in children < 5 years of age (10-12) and is associated with a reduction in the proportion of children with a serum bactericidal antibody (SBA) level above the accepted correlate of protection of ≥ 8 (Figure 2) (10, 11, 13). This is well illustrated in Figure 2 which shows MenC-PS specific IgG concentrations in the Dutch population decreasing to pre-immunization levels within a few years after primary vaccination at 14 months. This coincides with a decline in the percentage of children with SBA-levels above the correlate of protection. SBA-levels above the correlate of protection cut-off (titer≥8) are essential for sufficient protection against MenC, as invasion of MenC and subsequent devastating disease may occur within hours after acquisition of the organism into the nasopharynx, while a booster immune response in a previously primed individual normally takes days to develop.

In contrast to the children vaccinated during infancy, children vaccinated at older ages appear to have a better and longer-lasting antibody response than those vaccinated at a young age (10, 14).

Figure 2a outlines the MenC-PS specific IgG concentrations of the Dutch population in 1995/1996 (blue) and 2006/2007 (red). The red line between the vertical dashed lines outlines the MenC-PS specific IgG concentrations of the cohort vaccinated during the catch-up campaign in 2002 5 years after this vaccination. It shows an age-dependent rise in antibody response after primary vaccination: the higher the age at primary vaccination, the higher the remaining antibody level 5 years later. This coincides with a higher percentage of children with SBA levels above the correlate of protection cut-off (figure 2b).

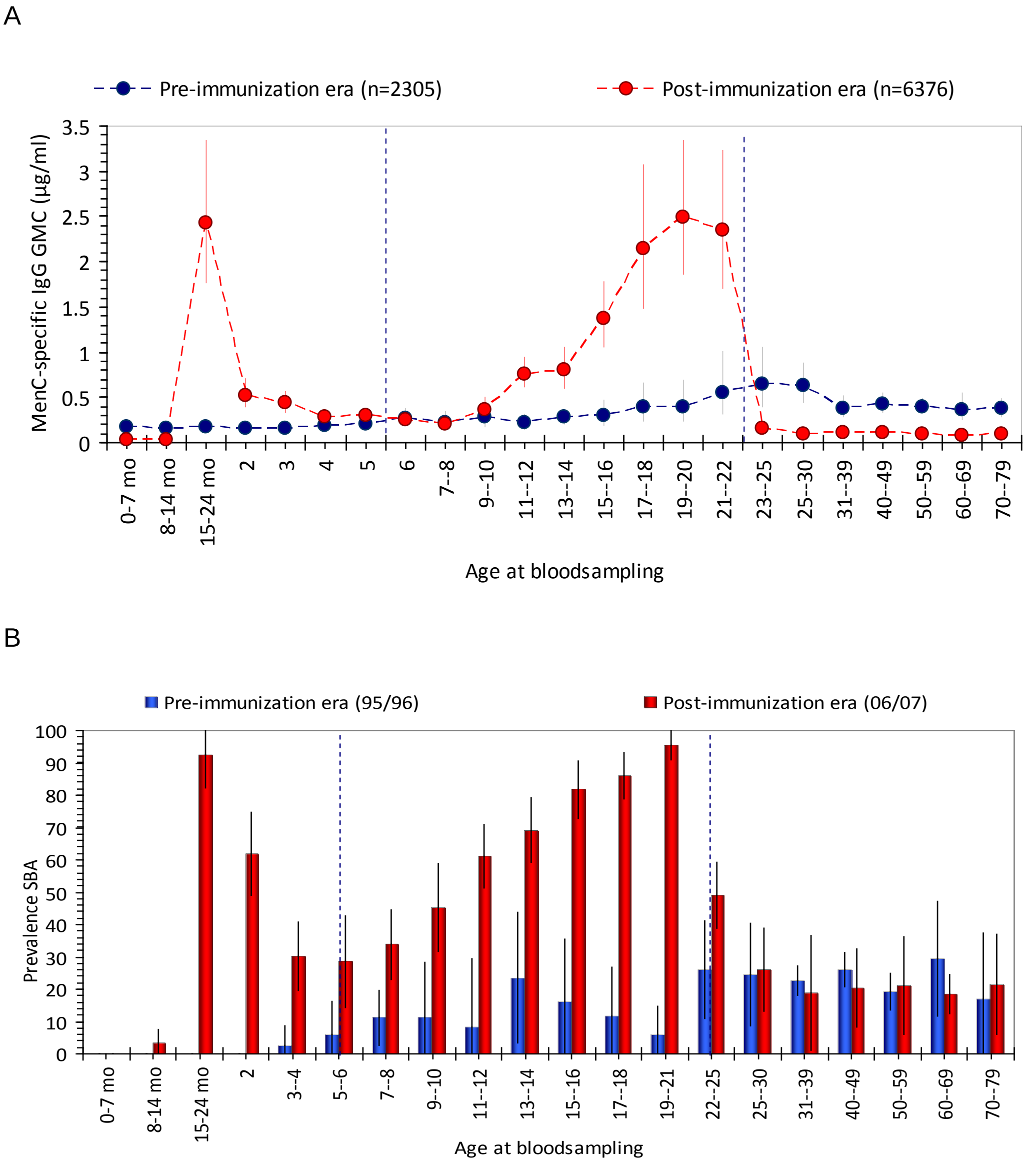

Figure 2. MenC-PS specific IgG and serum bactericidal antibody (SBA) levels in Dutch population. MenC PS-specific IgG (A) and seroprevalence of SBA titers ≥8 (B) within each age-cohort, pre- (1995/1996, in blue) and 5 years post (2006/2007, in red) introduction of the MenC conjugate vaccine in 2002. Error bars indicate 95% confidence intervals. Area between vertical lines indicates cohorts that were immunized in catch-up campaign in 2002. Age at blood sampling is stated in years unless indicated otherwise (mo = age in months)

As also shown in figure 2, introduction of MenCC vaccination in 2002 led to lowered MenC-PS specific IgG concentrations in the youngest (< 14 months) and older unvaccinated age-groups. This is probably caused by decreased circulation of MenC among the population due to the mass-vaccination which reduced the amount of natural infection. This poses these groups at risk when MenC starts recirculating in the community.

It is unknown how long the herd immunity for MenC disease will last. However, it is clear that the current single vaccination at 14 months is not sufficient to attain long standing protection against MenC and that there is a need for a second vaccination at an older age. Without a second vaccination with the MenCC vaccine, children vaccinated at 14 months will reach the second period of increased risk for invasive MenC disease (12-18 years) with low serologic markers of protective immunity. If MenC starts recirculating again, this might have devastating effects in both this cohort and all the unvaccinated individuals. Importantly, a second vaccination will not only prolong individual immunity but might also secure sustained herd immunity.

The aim of this study is to determine the appropriate age for a second MenC conjugate vaccine immunization. Determining the appropriate age depends on several factors. First of all, the appropriate age should mainly be based on the quantity (antibody concentrations, persistence of antibody levels) and quality (SBA titer, avidity) of the immune response to a second MenCC vaccination. Based on figure 2, an appropriate age for a second vaccination seems to be 15 years, since a primary vaccination at this age led to the highest MenC-PS specific IgG antibody and SBA levels 5 years later. However, before introduction of the MenCC vaccination the second peak of MenC invasive disease incidence was found between 12-18 years of age. It might therefore be sensible to administer the second vaccination at an earlier age to establish sufficient antibody levels before a child reaches adolescence. A second factor that determines the appropriate age is the feasibility of implementing a second MenCC vaccination into the NIP at that age. In the Dutch NIP, children are already offered a routine MMR and DT-IPV vaccination at the age of 9 years. In addition, Dutch girls are recently offered a HPV-vaccination at the age of 12 years (the year they turn 13). It would be convenient if the second MenCC vaccination would be implemented at one of these vaccination time points. On the other hand, immune responses against the vaccines routinely given to children aged 9 or 12 years might interfere with the immune response against the MenCC vaccine that will be given in this study (15). This can disturb a clear comparison of the quantity and quality of the immune response against a second MenCC vaccination between the three age groups. For this study, we decided to avoid potential interference of the immune response against NIP vaccines with the immune response against the MenCC vaccine. We therefore chose the age groups 10, 12* and 15 years to investigate the immune response to a second MenCC vaccination, in order to determine the appropriate age for a second MenCC vaccine.

*** In 2011 Dutch girls born in 1998 receive 3 HPV vaccinations: 2 in the spring and 1 in the fall. The TIM-study is scheduled to start in Octoberl 2011. Girls receiving their 3rd HPV vaccination at that time are mostly 13 years old. In order to avoid interference with HPV vaccination we therefore chose to include 12 year olds for this study.**

2. OBJECTIVES

The main purpose of this study is to determine the appropriate age (10, 12 or 15 years) for a second MenC conjugate (MenCC) vaccine immunization in Dutch children that received a primary MenCC vaccination at a young age. A conclusion will be based on the quality and quantity of the MenC-PS specific antibody response against a second MenCC vaccination at these different ages.

2.1 Primary objectives

- To assess SBA levels at T0 and at 1 month (T1) and 1 year (T2) after the second MenCC vaccination and determine whether there is a difference between the different age groups in the levels and the proportion of participants that have an SBA level of ≥8 (persistence of vaccine induced protective antibody levels).

2.2 Secondary objectives

- To assess serum MenC-PS specific IgG levels at T0 (prior to vaccination) and at 1 month (T1) and 1 year (T2) after the second MenCC vaccination and determine whether there is a difference in IgG levels between the different age groups (persistence of vaccine induced antibody levels)
- To assess avidity of serum MenC-PS specific IgG antibodies and determine whether there is a difference in avidity between the different age groups
- To assess whether there is a difference between the different age groups in the decay rate of MenC-PS specific antibody levels after secondary vaccination.
- To determine whether there is a difference in MenC-PS specific antibody subclasses (e.g. IgG1-4, IgG1/IgG2 ratio) between the different age groups.
- To determine whether there is a difference in avidity of MenC-PS specific IgG antibodies after primary versus secondary vaccination.
- To investigate longitudinal kinetics of B- and T- cell memory immune responses after primary and secondary MenCC vaccination (e.g. presence and functionality of memory B-cells and T-cells prior to and after the second MenCC vaccination).
- To measure serum IgG antibody levels against tetanus, the carrier protein for the MenC polysaccharide in the conjugate vaccine, to investigate the effect of a second MenCC vaccine on these titers.
- To measure salivary and serum IgA levels at T0, T1 and T2 in order to investigate their correlation and the (longitudinal) kinetics of local and systemic IgA production after primary and secondary MenCC vaccination. IgA is the major antibody at mucosal surfaces and considered to be important in limiting meningococcal colonisation and preventing early invasion.

3. STUDY DESIGN

This is an intervention study to determine the appropriate age for a second MenCC vaccination. The effect of a second MenCC vaccination will be investigated in three age-groups: 10-, 12- and 15 year olds.

3.1 Recruitment

The participants will be recruited from Utrecht (Leidsche Rijn) and the surrounding region of Utrecht (Maarsen, Vleuten, Nieuwegein, IJsselstein, Zeist, Bilthoven and Houten). Addresses from eligible children (based on their age at the start of the study) will be attained through the 'Regionale Coordinatie Programma's (RCP).' An invitation letter will be sent to the parents of all potential participants (Annex 1: *Invitation Letter*). This invitation letter includes brief information about the study and a reply card with the question whether or not the parents and child are willing to participate in the study and want more information. Based on former similar studies conducted by the RIVM, a participation percentage of 5-10% can be expected. We would like to include 82 participants per age group (see section 4.4 for sample size calculation). The invitation letter will therefore be sent to +/- 4000 potential participants in the above mentioned region

After receiving the reply card that indicates that a child and its parent(s) are willing to participate, the principal investigator will contact the (parents of the) child to give more information and to check whether the child is eligible for inclusion in the study based on the inclusion and exclusion criteria (see 4.2 and 4.3). Afterwards an extensive information letter (Annex 2: *Patient Information Letter*) together with an informed consent form (Annex 3: *Informed Consent*) will be sent to the potential participant. Within one week after sending the information, the principal investigator will contact the (parents of the) child a second time to answer additional questions. If parents and child remain willing to participate, an appointment is made for the first visit at a study site close to where the potential participant lives. During this first visit, the informed consent form will be signed by the principal investigator. The participant and his/her parents are asked to sign the informed consent form in advance to ensure that *both* parents signed the form. Afterwards, the study will start.

3.1.1 Study sites

In every city or village where participants are recruited, a local study site will be set up (e.g. local public health centers). This will ensure that the participants do not have to travel too far to visit the study site.

3.2 Vaccination and collection of blood and saliva samples

The total duration of the study is one year and comprises three visits from the participants to a study site. All participants will receive their second MenCC vaccination (they have been primed at an earlier age) at the first visit. Blood samples will be drawn prior to this second MenCC vaccination (T0) and 1 month (28-42 days, T1) and 1 year (T2) after the vaccination. In addition, saliva samples will be obtained at T0, T1 and T2. See Table 1 for an overview of the study schedule. The study will start as soon as possible and end one year later.

Table 1. Study calendar

Visit number at study site	Actions
T0: First visit	- sign informed consent form<br>- draw first blood sample(s)<br>- draw first saliva sample<br>- administer second MenCC vaccination
T1: Second visit (1 month after T0)	- draw second blood sample(s)<br>- draw second saliva sample
T2: Final visit (1 year after T0)	- draw final blood sample(s)<br>- draw final saliva sample

From most participants, one blood sample of 5 mL per visit will be drawn. This blood sample will be used to attain quantitative and qualitative information on the antibody response that is evoked by the second MenCC vaccination. In order to study cellular responses after MenCC vaccination, an additional 16 mL of blood is needed. A subset of the participants will be asked for permission for drawing this additional 16 mL (2 samples of 8 mL) of blood at all visits. This comes down to a total of three blood samples per visit instead of one. The aim is to obtain these additional samples from 20 of the participants per age group (total of 60).

4. STUDY POPULATION

4.1 Population (base)

Three age-groups will be recruited:

- Group 1: 10 years olds (male + female, n=82)
- Group 2: 12 year olds (male + female, n=82)
- Group 3: 15 year olds (male + female, n=82)

The participants will be recruited from Utrecht (Leidsche Rijn) and the surrounding region of Utrecht (Maarsen, Vleuten, Nieuwegein, IJsselstein, Zeist, Bilthoven and Houten).

4.2 Inclusion criteria

Participants are 10-, 12, and 15-year old children who have received a primary vaccination with a *single* dose of MenC-PS conjugated (MenCC) vaccine NeisVac-C™ either during the mass catch-up campaign in 2002 (group 2 and 3) or at the age of 14 months (regular vaccination time point since 2002 according to the Dutch NIP; group 1).

Furthermore, participants have to fulfil all of the following criteria:

- Provision of written informed consent by both parents and (if child is 12or 15 years old; see Annex 3) child;
- Good general health;
- Received all regular vaccines according to Dutch NIP
- Adherent to protocol, and available during the study period.

4.3 Exclusion criteria

Any of the following criteria at the start of the study will exclude a volunteering child from participation:

- Severe acute (infectious) illness or fever (>38.5°C) within 14 days before vaccination;
- Antibiotic use within 14 days of enrollment;
- Present evidence of serious disease(s) demanding medical treatment that might interfere the results of the study (chronic infection, bleeding disorder, immune dysfunction, genetic anomaly);
- Known or suspected allergy to any of the vaccine components (by medical history);
- Occurrence of serious adverse event after primary MenCC vaccination or other vaccination (by medical history)
- Known or suspected immune deficiency;
- History of any neurologic disorder, including epilepsy;
- Previous administration of plasma products (including immunoglobulins) within the last 6 months;
- Pregnancy.
- Previous confirmed or suspected meningococcal disease.
- Former received doses of MenC vaccines in addition to the primary vaccination
- Received vaccination in the past month

Presence of the in- and exclusion criteria (including pregnancy) will be checked by interviewing the parent about the medical history of the child during the first telephone call. In addition, presence of the exclusion criteria (including pregnancy) will be checked by interviewing the parent and child at the first study visit, prior to signing the informed consent form.

4.4 Sample size calculation

The sample size calculation outlined here is based on data from a comparable study that was recently performed in the UK by Perrett et al (16). We require a sample size that allows us to show a significant 2-fold difference in geometric mean SBA titers (SBA GMT) between one of the age groups (10, 12 r 15 years) and one or both other age groups. SBA GMT between groups will be tested in three independent <u>two-sided</u> t-tests as follows: 10 years vs 12years, 10 years vs 15 years and 12 years vs 15 years.

H_0: $GMT_{10} = GMT_{12}$, $GMT_{10} = GMT_{15}$, $GMT_{12} = GMT_{15}$

H_a: GMT_{10}<> GMT_{12}, GMT_{10} <> GMT_{15}, GMT_{12} <> GMT_{15}

In order to calculate the sample size, the following measures are needed:

- significance level (**α**): here α /nr of tests (Bonferroni correction for multiple testing) = 0.05/3
- desired power: here 0.80
- standard deviation (**σ**) of titers
- difference (**δ**) between 2 groups

<u>*Calculation of **σ***</u>

We have taken the sample sizes (n) and SBA GMT with 95%CI for 9-, 10-, 11- and 12-years olds from a comparable study by Perrett et al (16):

**Mean age (±SD)**	**n**	**SBA GMT**	**SBA GMT lower limit**	**SBA GMT upper limit**
9.26 (0.44)	28	551	337	902
10.27 (0.32)	25	605	406	901
11.30 (0.36)	26	705	512	970
12.09 (0.27)	26	1302	856	1978

We assume that log(GMT) is t-distributed with n-1 degrees of freedom (df). Its standard error (se) is then estimated by:

$$se[\log(GMT)] = \frac{\log(\text{upper limit}) - \log(\text{lower limit})}{2t_{(1-\alpha/2,\ n-1)}}$$

The standard deviation of the titer data is then given by:

$\sigma[\log(titer)] = se[\log(GMT)] * \sqrt{n}$

This leads to the values for σ[log(titer)] outlined in the table below:

Mean age (±SD)	n	SBA GMT	SBA GMT lower limit	SBA GMT upper limit	SE (log GMT)	σ (log titer)	log GMT
9.26 (0.44)	28	551	337	902	0.2399154	1.2695131	6.311735
10.27 (0.32)	25	605	406	901	0.1931180	0.9655902	6.405228
11.30 (0.36)	26	705	512	970	0.1551249	0.7909849	6.558198
12.09 (0.27)	26	1302	856	1978	0.2033395	1.0368321	7.171657

The 9-year olds have the largest standard deviation. To be sure, this standard deviation is used for the sample size calculation.

Calculation of ***δ***

We require a 2-fold difference in SBA GMT between 2 groups to be statistically significant. On a log-scale, the difference is given by:

$GMT_1 = 2*GMT_2$

$\log(GMT_1) = \log(2*GMT_2) = \log(2) + \log(GMT_2)$

$\delta = \log(GMT_1) - \log(GMT_2) = \log(2)$

Sample size

Using an α of (0.05/3), a power of 0.80, the σ[log(titer)] of the 9 year olds and a δ of log(2) in the sample size calculation leads to a sample size of 72 participants per group. Assuming that 10% of the participants will leave the study, and blood sampling will fail in +/- 2 participants per group, we aim to include 82 participants per group.

5. TREATMENT OF SUBJECTS

5.1 Investigational product/treatment

During this study the subjects will receive one dose of a vaccine that is routinely administrated to children in the NIP: NeisVac-C™(Registration number: RVG 26343). One dose of 0.5 ml for intramuscular injection contains 10 microgram of *Neisseria meningitidis* group C (strain C11) polysaccharide (de-O-acetylated). The polysaccharide is conjugated to 10-20 micrograms of tetanus toxoid and is adsorbed to aluminium hydroxide (0.5 mg). Other additive products are sodium chloride and water for injections.

5.2 Use of co-intervention

Not applicable

5.3 Escape medication

Not applicable

6. INVESTIGATIONAL MEDICINAL PRODUCT

6.1 Name and description of investigational medicinal product

During this study the subjects will receive one dose of NeisVac-C™(Registration number: RVG 26343). One dose of 0.5 ml for intramuscular injection contains 10 microgram of *Neisseria meningitidis* group C (strain C11) polysaccharide (de-O-acetylated). The polysaccharide is conjugated to 10-20 micrograms of tetanus toxoid and is adsorbed to aluminium hydroxide (0.5 mg). Other additive products are sodium chloride and water for injections.

6.2 Summary of findings from non-clinical studies

See Investigators Brochure (Annex 4) page 6-8 of NeisVac-C™.

6.3 Summary of findings from clinical studies

See Investigators Brochure (Annex 4) page 6-8 of NeisVac-C™ and reference (10, 17).

6.4 Summary of known and potential risks and benefits

See patient information leaflets (Annex 5) and Investigators Brochure (Annex 4) of NeisVac-C™.

6.5 Description and justification of route of administration and dosage

The NeisVac-C™ vaccine (0.5 ml) is injected intramuscularly in the upper arm. This is a customary and well-accepted route of administration of this vaccine. See page 2 of Investigators Brochure.

6.6 Dosages, dosage modifications and method of administration

All study subjects will receive one intramuscular injection with one dosage of the vaccine NeisVac-C™.

6.7 Preparation and labelling of Investigational Medicinal Product

See Investigators Brochure (Annex 4), page 9 and the SOP for vaccination (Annex 6)

6.8 Drug accountability

Vaccines will be provided by Baxter. Vaccines are stored and transported at 2-8˚C (see page 8 of Investigators Brochure (Annex 4)).

7. METHODS

7.1 Study parameters/endpoints

Blood and saliva samples will be collected from all participants at three time points: prior to the second MenCC vaccination (T0) and 1 month (T1) and 1 year afterwards (T2). Saliva supernatants, serum and PBMCs will be isolated from these samples in order to determine the following parameters:

7.1.1 Main study parameters

7.1.1.1 Serum MenC-PS specific IgG antibodies, -subclasses and –avidity

In order to achieve the primary and secondary objectives, geometric mean concentrations, subclasses and avidity of MenC polysaccharide (PS) specific IgG antibodies will be determined.

7.1.1.2 Serum Bactericidal Antibody assay (SBA) levels

SBA levels are a measure for MenC functional antibody activity. An SBA level of ≥ 8 is considered as a good correlate of protection against invasive MenC disease (18). SBA levels are expressed as the reciprocal of the final serum dilution yielding ≥ 50% killing at 60 minutes (10). In addition, the geometric mean titers (GMT) of SBAs will be determined and used for comparison between groups (see section 9.1 and 9.2).

7.1.2 Secondary study parameters

7.1.2.1 MenC specific B-cell and T-cell responses

Part of the participants will be asked for an additional 16 mL of blood for the purpose of studying cellular responses (see section 3.2). This blood will be collected in vacutainer cell preparation tubes (CPT) and used for the isolation of PBMCs (17). PBMCs will be divided in purified B- cell populations and T- cell populations. B-cells will be cultured and memory B- cells will be polyclonally stimulated (19). After stimulation, B- cell memory responses will be measured against MenC-PS. T-cell cultures will be stimulated with tetanus toxoid (TT), TT-MenC conjugate without alum or MenC PS. After stimulation, detection of IFN-γ secreting T-cells will be performed (4, 20).

7.1.2.2 Serum and salivary MenC-PS specific IgA

IgA is the major antibody at mucosal surfaces and considered to be important in limiting meningococcal colonisation and preventing early invasion. Serum and salivary MenC-PS specific IgA levels will be measured in order to investigate their correlation and the (longitudinal) kinetics of local and systemic IgA production after primary and secondary MenCC vaccination.

7.1.3 Other study parameters

During the course of the study it could be possible that additional parameters turn out be of interest, such as antibody levels other than the ones mentioned here, or certain cytokine levels. These parameters will then be measured using the most suitable laboratory tests available at the RIVM. If newly developed and better laboratory tests become available, these will be used wherever possible.

7.2 Randomisation, blinding and treatment allocation

Not applicable

7.3 Study procedures

7.3.1 Invasive study procedures

The invasive study procedures (vaccination and venapunctures) will be carried out by experienced and qualified persons according to standard operating procedures. A local anesthetic (Xylocaine spray) can be used to minimize the pain of the venapuncture.

7.3.1.1 Vaccination

See SOP of vaccination (Annex 6)

7.3.1.2 Venapuncture

See SOP of venapuncture (Annex 7)

7.3.1.3 Saliva sampling

See SOP of saliva sampling (Annex 8)

7.3.2 Laboratory tests

7.3.2.1 Fluorescent-bead-based multiplex immunoassay (MIA)

The fluorescent-bead-based multiplex immunoassay (MIA) will be used to measure anti-MenC-PS specific IgG antibody concentrations using CDC1992 reference serum as standard. See reference (21) for the detailed procedure. The MIA will also be used to measure MenC-PS specific IgG subclasses and avidity (17, 21) to measure serum and salivary IgA and to measure IgG antibody levels against tetanus, the carrier protein for the MenC polysaccharide in the conjugate vaccine (22). Finally, the MIA will also be used to measure cytokine levels from the B- and T-cell supernatants.

7.3.2.2 Serum Bactericidal Antibody (SBA) assay

The serum bactericidal antibody assay will be used for measurement of MenC SBA levels, using baby rabbit complement (23) and the O-acetylated serogroup C strain

C11 (phenotype C:16:P1.7-1,1). SBA titers are expressed as the reciprocal of the final serum dilution yielding ≥ 50% killing at 60 minutes (10) and as geometric mean titers (GMT).

7.3.2.3 Avidity assay

To assess avidity of MenC-PS specific IgG antibodies, serum samples will be incubated with ammonium thiocyanate (NH_4SCN, 0.5M), in order to dissociate low-avidity antigen-antibody binding, or with PBS. See reference (17) for the procedure.The level of avidity of IgG antibodies will be expressed as the avidity index (AI). This is the percentage of IgG antibodies that remains bound to MenC PS-conjugated beads after treatment with NH_4SCN (measured with MIA) and is calculated as follows:

$$AI = \frac{\text{(IgG concentration after incubation with } NH_4SCN\text{)}}{\text{(IgG concentration after incubation with PBS)}} \times 100\%$$

An AI of 0-33% is arbitrarily indicated as *low*, 33-66% as *intermediate* and 66-100% as *high* (4, 24).

7.3.2.4 ELIspot

B-cell memory responses against MenC-PS will be measured by ELIspot assays. See reference (17) for the procedure. T-cell cultures will be stimulated with tetanus toxoid (TT), TT-MenC conjugate without alum or MenC PS. After stimulation, detection of IFN-γ secreting T-cells will also be done through ELIspot assays (4, 20).

7.4 Withdrawal of individual subjects

Subjects can leave the study at any time for any reason if they wish to do so without any consequences. The investigator can decide to withdraw a subject from the study for urgent medical reasons.

7.5 Replacement of individual subjects after withdrawal

Not applicable.

7.6 Follow-up of subjects withdrawn from treatment

Not applicable.

7.7 Premature termination of the study

NeisVac-C™ is a registered vaccine which has already been used in the Netherlands (and other countries) in the same age group (children 10, 12 and 15 years of age), e.g. in the catch-up

campaign in 2002. It is therefore unlikely that serious side effects will occur that can lead to premature termination of the study.

8. SAFETY REPORTING

8.1 Section 10 WMO event

In accordance to section 10, subsection 1, of the WMO, the investigator will inform the participants and the reviewing accredited METC if anything occurs, on the basis of which it appears that the disadvantages of participation may be significantly greater than was foreseen in the research proposal. The study will be suspended pending further review by the accredited METC, except insofar as suspension would jeopardise the participants' health. The investigator will take care that all participants are kept informed.

8.2 Adverse and serious adverse events

Adverse events (AEs) are defined as any undesirable experience occurring to a participant during the study, whether or not considered related to the vaccine, the vaccination or the venapuncture. All adverse events reported spontaneously by the participant, his/her parent(s) or observed by the principal investigator or her staff will be recorded in the case report form (CRF Annex 9).

A serious adverse event (SAE) is any untoward medical occurrence or effect that at any dose of the vaccine:

- results in death;
- is life threatening (at the time of the event);
- requires hospitalisation or prolongation of existing inpatients' hospitalisation;
- results in persistent or significant disability or incapacity;
- is a new event of the trial likely to affect the safety of the participant, such as an unexpected outcome of an adverse reaction, major safety finding from a newly completed animal study, etc.

SAEs will lead to definite suspension of the study participant.

All SAEs will be reported by the principal investigator through the web portal *ToetsingOnline* to the accredited METC that approved the protocol, within 15 days after the sponsor has first knowledge of the serious adverse reactions. SAEs that result in death or are life threatening should be reported expedited. The expedited reporting will occur not later than 7 days after the principal investigator has first knowledge of the adverse reaction. This is for a preliminary report with another 8 days for completion of the report.

All SAEs with a suspected (probable or definite) relationship to the vaccine (as indicated by the responsible investigator) will be reported to Lareb and the Medicines Evaluation Board (CBG) by the sponsor.

8.2.1 Suspected unexpected serious adverse reactions (SUSAR)

Adverse reactions are all untoward and unintended responses to an investigational product related to any dose administered.

Unexpected adverse reactions are adverse reactions, of which the nature, or severity, is not consistent with the applicable product information (e.g. Investigator's Brochure for an unapproved IMP or Summary of Product Characteristics (SPC) for an authorised medicinal product).

The sponsor will report expedited the following SUSARs through the web portal *ToetsingOnline* to the METC:

- SUSARs that have arisen in the clinical trial that was assessed by the METC;
- SUSARs that have arisen in other clinical trials of the same sponsor and with the same medicinal product, and that could have consequences for the safety of the subjects involved in the clinical trial that was assessed by the METC.

The remaining SUSARs are recorded in an overview list (line-listing) that will be submitted once every half year to the METC. This line-listing provides an overview of all SUSARs from the study medicine, accompanied by a brief report highlighting the main points of concern.

The expedited reporting of SUSARs through the web portal ToetsingOnline is sufficient as notification to the competent authority.

The sponsor will report expedited all SUSARs to the competent authorities in other Member States, according to the requirements of the Member States.

The expedited reporting will occur not later than 15 days after the sponsor has first knowledge of the adverse reactions. For fatal or life threatening cases the term will be maximal 7 days for a preliminary report with another 8 days for completion of the report.

All SUSARs will also be reported to the CBG and the distributor of the vaccine.

8.2.2 Annual safety report

Not applicable. NeisVac-C™ is a registered vaccine and currently used in the Dutch NIP for all children aged 14 months. Furthermore, the vaccine has already been used in the Netherlands (and other countries) in the same age group (children 10, 12 and 15 years of age) during the catch-up campaign in 2002. During the catch-up campaign over 3 million children were vaccinated and only 1512 developed and adverse reaction. All adverse reactions are described in the RIVM rapport 240082001/2004

'*Ervaringen met bijwerkingen van de eenmalige Meningokokken C-vaccinatiecampagne in 2002.*' Of the 1512 adverse reactions reported, 41 were considered serious. All these 41 children recovered completely and the mass vaccination campaign with NeisVac-C™ was described as extremely safe. We therefore consider it very unlikely that new (serious) adverse reactions will occur that are not already described in the Investigators Brochure.

8.3 Follow-up of adverse events

All adverse events will be followed until they have abated, or until a stable situation has been reached. Depending on the event, follow up may require additional tests or medical procedures as indicated, and/or referral to the general physician or a medical specialist.

8.4 Data Safety Monitoring Board (DSMB)

Not applicable.

9. STATISTICAL ANALYSIS

Data analysis will be performed by the principal and sponsor investigators. Validated data sets will be used for analysis.

9.1 Descriptive statistics

All antibody concentrations will be log-transformed for the analyses. Results will be expressed in geometric mean concentrations (GMC). SBA levels of the groups at different time points will be expressed in geometric mean titers (GMT). SBA levels will be expressed as the reciprocal of the final serum dilution yielding ≥ 50% killing at 60 minutes (10). An SBA level of ≥ 8 is considered as a good correlate of protection (18). Analysis of differences at T0, T1 and T2 in the proportion of participants in each group that have a SBA-level of ≥8 will be descriptive.

The level of avidity of MenC-PS specific IgG antibodies will be expressed as the avidity index (AI). This is the percentage of IgG antibodies that remains bound to MenC PS-conjugated beads after treatment with NH_4SCN and is calculated as follows:

$$AI = \frac{\text{(IgG concentration after incubation with } NH_4SCN\text{)}}{\text{(IgG concentration after incubation with PBS)}} \times 100\%$$

An AI of 0-33% is arbitrarily indicated as *low*, 33-66% as *intermediate* and 66-100% as *high (4, 24)*.

Analysis of the presence and functionality of memory B-cells and T-cells prior to and after the second MenCC vaccination will be descriptive.

9.2 Univariate analysis

Statistical analysis will be carried out with the SPSS version 19.0.0 (SPSS inc, Chicago, Illinois, USA). Limit values for significance will be set at 0.05 for all 2-sided tests performed. Confidence intervals (CI) will be expressed as 95%-CI.

To determine whether serum MenC-PS specific IgG levels, SBA levels, serum IgA or salivary IgA levels at T0, T1 and T2 differ between the age groups, the geometric mean concentrations (GMC) of MenC-PS specific IgG, IgA and the SBA geometric mean titers (GMT) will be tested in independent <u>two-sided</u> t-tests as follows: 10 years vs 12 years, 10 years vs 15 years and 12 years vs 15 years.

H_0: $GMT_{10} = GMT_{12}$, $GMT_{10} = GMT_{15}$, $GMT_{12} = GMT_{15}$

H_a: $GMT_{10} <> GMT_{12}$, $GMT_{10} <> GMT_{15}$, $GMT_{12} <> GMT_{15}$

(ANOVA is not suitable here, as this test will only tell us whether there is *any* difference between the 3 groups. It will not tell us which group differs from the others, nor the size of this difference.)

To determine whether there is a difference in avidity of IgG antibodies between the different age-groups at T1 and T2, the proportion of participants with a high (or low) AI will be compared using χ^2-tests.

To determine whether there is a difference in antibody subclasses (IgG1-4) between the different age groups at T0, T1 and T2, GMCs will be tested in two-sided independent t-tests (assuming log-transformation of these titers leads to an approximate normal distribution). Analysis of differences in IgG1/IgG2 ratio between the groups will be descriptive.

Difference in the AI of MenC-PS specific IgG antibodies after primary versus second vaccination within the groups will be tested using χ^2-tests.

To determine whether serum and saliva levels of MenC specific IgA are correlated, Pearsons correlation coefficients will be computed.

A possible confounder in the above mentioned analyses could be the antibody and SBA levels at baseline (T0). However, we assume that antibody levels in all three the age groups will be decreased to unprotective pre-vaccination levels at baseline and that there will be no statistical differences in these levels between the three groups at baseline. If there appears to be a difference in these levels at baseline, all other analyses will be adjusted for the level at baseline using linear regression analyses.

9.3 Multivariate analysis

Not applicable.

9.4 Interim analysis (if applicable)

Not applicable.

10. ETHICAL CONSIDERATIONS

10.1 Regulation statement

This clinical study will be performed according to the current rules for Good Clinical Practice (GCP), as described by the Committee for Proprietary Medical Products (CPMP) of the European Union and the International Committee on Harmonization (ICH) in "Note for Guidance on Good Clinical Practice, document CPMP/ICH/135/95", effective since January 17th 1997 and according to the Dutch Medical Research Involving Human Subjects Act (WMO), under the general ruling of the Clinical Trial Directive of the EU (2001/20/EU). These rules include the ethical guidelines described in the "Declaration of Helsinki" (World Medical Association Declaration of Helsinki: 'Ethical Principles for Medical Research involving Human Subjects'. Adopted by the 18th World Medical Association (WMA), Helsinki, Finland, 1964; amended by the 29th WMA, Tokyo, Japan, 1975; 35th WMA, Venice, Italy, 1983; 41st WMA, Hong Kong, 1989; the 48th WMA, Sommerset West, Republic of South Africa, 1996; 52nd WMA, Edinburgh, Scotland, 2000; 53rd WMA Washington, USA, 2002; 55th WMA Tokyo, Japan, 2004 and the 59th WMA General Assembly in Seoul, 2008).

10.2 Recruitment and informed consent

The participants will be recruited from Utrecht (Leidsche Rijn) and the surrounding region of Utrecht (Maarsen, Vleuten, Nieuwegein, IJsselstein, Zeist, Bilthoven and Houten). Addresses from eligible children (based on their age at the start of the study) will be attained through the 'Regionale Coordinatie Programma's (RCP).' An invitation letter will be sent to the parents of all potential participants (Annex 1: *Invitation Letter*). This invitation letter includes brief information about the study and a reply card with the question whether or not the parents and child are willing to participate in the study and want more information. Based on former similar studies conducted by the RIVM, a participation percentage of 5-10% can be expected. The invitation letter will therefore be sent to +/-4000 potential participants in the above mentioned region.

After receiving the reply card that indicates that a child and its parent(s) are willing to participate, the principal investigator will contact the (parents of the) child to give more information and to check whether the child is eligible for inclusion in the study based on the inclusion and exclusion criteria. Afterwards an extensive information letter (Annex 2: *Patient Information Letter*) together with an informed consent form (Annex 3: *Informed Consent*) will be sent to the potential participant. Within one week after sending the information, the principal investigator will contact the (parents of the) child a second time to answer additional questions. If parents and child are willing to participate, an appointment is made for the first visit at a study site close to where the potential participant lives. During this first visit, the informed consent form will be signed by the principal investigator. The participant and his/her parents are asked to sign the informed consent form in advance to ensure that ***both*** parents signed the form. Afterwards, the study will start.

10.3 Objection by minors

All participants are minors. If during the course of the study one of the participants objects to voluntary participation to (one of) the study procedures (e.g. vaccination of venapuncture), the code ‘gedragscode verzet minderjarigen’ (WMO, Article. 4, lid 1) will be followed.

10.4 Benefits and risks assessment

Participants benefit from participating in the study by receiving an additional MenCC vaccination which theoretically provides increased protection against MenC invasive disease. This second MenCC vaccination is currently not routinely administered to these age-groups according to the NIP. From the public health perspective, participation in this study will contribute to the improvement of the National Immunisation Programme (NIP).

Vaccination and venapunctures might be painful and unpleasant. Nonetheless, they are relatively low risk invasive procedures. NeisVac-C™ is a registered vaccine in the Netherlands. Mild adverse reactions to the vaccine may occur but they are expected to be mainly local and transient (see Investigators Brochure Annex 4 page 5). Severe allergic reactions to one of the vaccine components are unlikely to occur; the chance of such an event to occur will reasonably not be larger than found after injection of other vaccines. The vaccine will only be used in the study if released by the manufacturer and the appropriate authorities. Furthermore, local discomforts may occur as a result of the performed invasive procedures. On request of the participant, Xylocaine spray can be used to reduce possible local pain during the venapuncture. As a compensation for the vaccination, the venapunctures and the saliva sampling, all participants will receive a total of €25,- in vouchers after completion of the study (see section 10.6).

10.5 Compensation for injury

According to a Ministerial Order, RIVM is excluded from compulsory insurance for clinical research as determined by the Dutch law on Medical Investigations (WMO, section 7, paragraph 6). Participants can recover the loss from RIVM. Any claims will be settled according to the terms of an insurance company. Participants will be informed about these terms in detail in the Patient Information Letter (see Annex 2).

10.6 Incentives

Participants will receive a voucher of €10,- after the second blood sampling and an additional voucher of €15,- after the last blood sampling.

11. ADMINISTRATIVE ASPECTS AND PUBLICATION

11.1 Handling and storage of data and documents

All study data will be registered in a collection of files (the source documents) and handled confidentially. Separate records will be made for each participant. Parents and child can declare whether they wish to receive a report on the level of protection the child developed after the second MenCC vaccination. These reports will be provided by the principal investigator. The principal investigator assures that the anonymity of the participants is maintained; keeping separate files of codes, names and addresses of participants. All clinical data on participants obtained from the handling, treatment (vaccination and venapuncture) and observation will be recorded on Case Report Forms (CRF Annex 9). The CRF forms the basis for further analysis of the study results. Personal identifiers will not be recorded on the CRF, with exception of date of birth, initials and gender. In addition, each participant is assigned a unique code (UTN number). To enable efficient data analysis, electronic data files will be created. Source documents and hard copies of electronic files/analyses will be stored according to GCP guidelines (for a period of 15 years if permission is obtained in the informed consent form).

11.1.1 Case Report Form

The CRF will contain information obtained according to the study assessments described in the previous chapters. The sponsor investigator is not entitled to know personal data of the participant. Thus, the principal clinical investigator is required to separate personal and study data on the CRF. Throughout the CRF, on every page, the UTN number will be used as the unique participant identifier. Furthermore, date and time of study procedures and assessments will be recorded throughout the CRF for all recorded observations (see Annex 9) .

11.1.2 Data Entry Procedures

Data registered in the source documents will be made available for analysis in electronic data files. To establish a validated data set for analysis, a procedure of entry and verification will be used. All necessary changes and corrections after data entry will be motivated, dated and signed by the investigators.

11.2 Amendments

A ‘substantial amendment’ is defined as an amendment to the terms of the METC application, or to the protocol or any other supporting documentation, that is likely to affect to a significant degree:

- the safety or physical or mental integrity of the subjects of the trial;
- the scientific value of the trial;
- the conduct or management of the trial; or
- the quality or safety of any intervention used in the trial.

All substantial amendments will be notified to the accredited METC and to the competent authority.

Non-substantial amendments will not be notified to the accredited METC and the competent authority, but will be recorded and filed by the sponsor.

11.2.1 Change of exclusion criterium

In the initial protocol (version 2 April 1 2011) 'past vaccination against Hepatitis B' was an exclusion criterion in order to attain a study group with a similar vaccination history. However, during recruitment of participants it appeared that many children received vaccinations against hepatitis A and B, because they travelled abroad with their parents for the holiday. It was therefore decided to include all children that received vaccinations in addition to the vaccinations of the NIP, as long as these vaccinations were not given within 1 month prior to the start of the study. See paragraph 4.3.

11.3 Annual progress report

The investigator/sponsor will submit a summary of the progress of the trial to the accredited METC once a year. Information will be provided on the date of inclusion of the first subject, numbers of subjects included and numbers of subjects that have completed the trial, serious adverse events/ serious adverse reactions, other problems, and amendments.

11.4 End of study report

The principal investigator/sponsor will notify the accredited METC of the end of the study within a period of 8 weeks. The end of the study is defined as the last patient's last visit.

In case the study is ended prematurely, the investigator will notify the accredited METC, including the reasons for the premature termination.

Within one year after the end of the study, the principal investigator/sponsor will submit a final study report with the results of the study, including any publications/abstracts of the study, to the accredited METC.

11.5 Public disclosure and publication policy

The study results will be reported in an internal report and submitted for publication in peer-reviewed journals. Publications will be drafted by the sponsor investigators.

12. REFERENCES

1. Stephens DS, Greenwood B, Brandtzaeg P. Epidemic meningitis, meningococcaemia, and Neisseria meningitidis. Lancet. 2007 Jun 30;369(9580):2196-210.
2. Sim RJ, Harrison MM, Moxon ER, Tang CM. Underestimation of meningococci in tonsillar tissue by nasopharyngeal swabbing. Lancet. 2000 Nov 11;356(9242):1653-4.
3. Caugant DA, Maiden MC. Meningococcal carriage and disease--population biology and evolution. Vaccine. 2009 Jun 24;27 Suppl 2:B64-70.
4. de Voer RM. Meningococcal C specific immune responses. Immunity in an era of immunization with conjugate vaccine. Utrecht: University of Utrecht/ RIVM; 2010.
5. Harrison LH, Trotter CL, Ramsay ME. Global epidemiology of meningococcal disease. Vaccine. 2009 Jun 24;27 Suppl 2:B51-63.
6. van der Ende A, Spanjaard L. Bacterial Meningitis in the Netherlands; Annual Report 2009. Amsterdam: Netherlands Reference Laboratory for Bacterial Meningitis (AMC/RIVM)2009.
7. Miller E, Salisbury D, Ramsay M. Planning, registration, and implementation of an immunisation campaign against meningococcal serogroup C disease in the UK: a success story. Vaccine. 2001 Oct 15;20 Suppl 1:S58-67.
8. Campbell H, Borrow R, Salisbury D, Miller E. Meningococcal C conjugate vaccine: the experience in England and Wales. Vaccine. 2009 Jun 24;27 Suppl 2:B20-9.
9. Maiden MC, Ibarz-Pavon AB, Urwin R, Gray SJ, Andrews NJ, Clarke SC, et al. Impact of meningococcal serogroup C conjugate vaccines on carriage and herd immunity. J Infect Dis. 2008 Mar 1;197(5):737-43.
10. de Voer RM, Mollema L, Schepp RM, de Greeff SC, van Gageldonk PG, de Melker HE, et al. Immunity against Neisseria meningitidis serogroup C in the Dutch population before and after introduction of the meningococcal c conjugate vaccine. PLoS One. 2010;5(8):e12144.
11. Snape MD, Kelly DF, Green B, Moxon ER, Borrow R, Pollard AJ. Lack of serum bactericidal activity in preschool children two years after a single dose of serogroup C meningococcal polysaccharide-protein conjugate vaccine. Pediatr Infect Dis J. 2005 Feb;24(2):128-31.
12. Trotter CL, Andrews NJ, Kaczmarski EB, Miller E, Ramsay ME. Effectiveness of meningococcal serogroup C conjugate vaccine 4 years after introduction. Lancet. 2004 Jul 24-30;364(9431):365-7.
13. Borrow R, Goldblatt D, Andrews N, Southern J, Ashton L, Deane S, et al. Antibody persistence and immunological memory at age 4 years after meningococcal group C conjugate vaccination in children in the United kingdom. J Infect Dis. 2002 Nov 1;186(9):1353-7.
14. Snape MD, Kelly DF, Salt P, Green S, Snowden C, Diggle L, et al. Serogroup C meningococcal glycoconjugate vaccine in adolescents: persistence of bactericidal antibodies and kinetics of the immune response to a booster vaccine more than 3 years after immunization. Clin Infect Dis. 2006 Dec 1;43(11):1387-94.
15. Dagan R, Poolman J, Siegrist CA. Glycoconjugate vaccines and immune interference: A review. Vaccine. 2010 Aug 2;28(34):5513-23.
16. Perrett KP, Winter AP, Kibwana E, Jin C, John TM, Yu LM, et al. Antibody persistence after serogroup C meningococcal conjugate immunization of United Kingdom primary-school children in 1999-2000 and response to a booster: a phase 4 clinical trial. Clin Infect Dis. 2010 Jun 15;50(12):1601-10.
17. de Voer RM, van der Klis FR, Engels CW, Schepp RM, van de Kassteele J, Sanders EA, et al. Kinetics of antibody responses after primary immunization with meningococcal serogroup C conjugate vaccine or secondary immunization with either conjugate or polysaccharide vaccine in adults. Vaccine. 2009 Nov 23;27(50):6974-82.
18. Frasch CE, Borrow R, Donnelly J. Bactericidal antibody is the immunologic surrogate of protection against meningococcal disease. Vaccine. 2009 Jun 24;27 Suppl 2:B112-6.
19. Bernasconi NL, Traggiai E, Lanzavecchia A. Maintenance of serological memory by polyclonal activation of human memory B cells. Science. 2002 Dec 13;298(5601):2199-202.

20. de Voer RM, Sanders EA, Engels CW, Kerkhof J, Rijkers GT, Buisman AM, et al. Cellular immunity after immunization with meningococcal serogroup C conjugate or polysaccharide vaccine in adults. Unpublished work.
21. de Voer RM, van der Klis FR, Engels CW, Rijkers GT, Sanders EA, Berbers GA. Development of a fluorescent-bead-based multiplex immunoassay to determine immunoglobulin G subclass responses to Neisseria meningitidis serogroup A and C polysaccharides. Clin Vaccine Immunol. 2008 Aug;15(8):1188-93.
22. van Gageldonk PG, van Schaijk FG, van der Klis FR, Berbers GA. Development and validation of a multiplex immunoassay for the simultaneous determination of serum antibodies to Bordetella pertussis, diphtheria and tetanus. J Immunol Methods. 2008 Jun 1;335(1-2):79-89.
23. Maslanka SE, Gheesling LL, Libutti DE, Donaldson KB, Harakeh HS, Dykes JK, et al. Standardization and a multilaboratory comparison of Neisseria meningitidis serogroup A and C serum bactericidal assays. The Multilaboratory Study Group. Clin Diagn Lab Immunol. 1997 Mar;4(2):156-67.
24. Meerveld-Eggink A, de Weerdt O, de Voer RM, Berbers GA, van Velzen-Blad H, Vlaminckx BJ, et al. Impaired antibody response to conjugated meningococcal serogroup C vaccine in asplenic patients. Eur J Clin Microbiol Infect Dis. 2010 Dec 24.
